# The incidence of cerebral arterial vasospasm following aneurysmal subarachnoid haemorrhage: a systematic review and meta-analysis

**DOI:** 10.1007/s00234-022-03004-w

**Published:** 2022-07-07

**Authors:** Lachlan Donaldson, Ashleigh Edington, Ruan Vlok, Inez Astono, Tom Iredale, Oliver Flower, Alice Ma, Keryn Davidson, Anthony Delaney

**Affiliations:** 1 Malcolm Fisher Department of Intensive Care Medicine, Royal North Shore Hospital, Reserve Rd, St. Leonards, Sydney, NSW 2065 Australia; 2 Division of Critical Care, The George Institute for Global Health, Faculty of Medicine UNSW, Sydney, Australia; 3 Northern Clinical School, Sydney Medical School, University of Sydney, Sydney, Australia; 4 Department of Neurosurgery, Royal North Shore Hospital, Sydney, Australia

**Keywords:** Subarachnoid haemorrhage, Intracranial vasospasm, Delayed cerebral ischaemia, Delayed neurological deterioration

## Abstract

**Purpose:**

To describe a pooled estimated incidence of cerebral arterial vasospasm (aVSP) following aneurysmal subarachnoid haemorrhage (aSAH) and to describe sources of variation in the reported incidence.

**Methods:**

We performed a systematic review and meta-analysis of randomised clinical trials (RCTs) and cohort studies. The primary outcome was the proportion of study participants diagnosed with aVSP. We assessed for heterogeneity based on mode of imaging, indication for imaging, study design and clinical characteristics at a study level.

**Results:**

We identified 120 studies, including 19,171 participants. More than 40 different criteria were used to diagnose aVSP. The pooled estimate of the proportion of patients diagnosed with aVSP was 0.42 (95% CI 0.39 to 0.46, *I*^2^ = 96.5%). There was no evidence that the incidence aVSP was different, nor that heterogeneity was reduced, when the estimate was assessed by study type, imaging modalities, the proportion of participants with high grade CT scores or poor grade clinical scores. The pooled estimate of the proportion of study participants diagnosed with aVSP was higher in studies with routine imaging (0.47, 95% CI 0.43 to 0.52, *I*^2^ = 96.5%) compared to those when imaging was performed when indicated (0.30, 95% CI 0.25 to 0.36, *I*^2^ = 94.0%, *p* for between-group difference < 0.0005).

**Conclusion:**

The incidence of cerebral arterial vasospasm following aSAH varies widely from 9 to 93% of study participants. Heterogeneity in the reported incidence may be due to variation in the criteria used to diagnose aVSP. A standard set of diagnostic criteria is necessary to resolve the role that aVSP plays in delayed neurological deterioration following aSAH.

**PROSPERO registration:**

CRD42020191895

**Supplementary Information:**

The online version contains supplementary material available at 10.1007/s00234-022-03004-w.

## Introduction

Although aneurysmal subarachnoid haemorrhage (aSAH) accounts for only 5% of all patients with acute stroke, with a peak incidence in a younger population, it accounts for the same proportion of life-years lost as acute ischaemic stroke [1]. Up to 75% of survivors of aSAH will be left with significant neurological morbidity [2]. These neurological sequelae are related to the early brain jury that results from the initial haemorrhage, as well as secondary or delayed neurological deterioration due to delayed cerebral ischaemia [3, 4]. Delayed cerebral ischaemia (DCI) has a standard consensus definition: the occurrence of focal neurological impairment or a decrease of at least 2 points on the Glasgow Coma Scale, which lasts for at least 1 h, is not apparent immediately after aneurysm occlusion and cannot be attributed to other causes by means of clinical assessment, CT or MRI scanning of the brain and appropriate laboratory studies [5]. It is known that DCI is a major factor contributing to neurological morbidity in patients who survive the initial haemorrhage [6].

The exact pathophysiology of DCI is not fully characterised [6]. Cerebral arterial vasospasm (aVSP), arterial narrowing demonstrated on imaging of the proximal cerebral vasculature, has been considered to play a role in the development of DCI [7], although more recent evidence suggests that other mechanisms may be more important. The contribution of aVSP to DCI and neurological outcome for patients following aSAH has not been resolved, with current guidelines for the management of patients following aneurysmal subarachnoid haemorrhage recommending screening for aVSP and specific interventions for the treatment of aVSP, all based upon low-level evidence [8, 9]. Clinical practice remains highly variable between centres and regions regarding the screening for aVSP and the use of medical and endovascular rescue techniques to treat patients with aVSP following aSAH [10, 11]. Treatment for patients with DCI and aVSP remains one of the most controversial areas in the management of patients with aSAH [4].

A major impediment to resolving the contribution of aVSP to DCI and delayed neurological deterioration following aSAH is the variation in the reported incidence of aVSP [5]. Therefore, we performed a systematic review and meta-analysis to address the question: In patient’s following aneurysmal subarachnoid haemorrhage, what is the estimated incidence of cerebral arterial vasospasm, and what are the sources of variation in the reported incidence?

## Methods

This review was undertaken according to a pre-published protocol (PROSPERO registration CRD42020191895) and is reported in accordance with the PRISMA statement [12].

### Eligibility criteria

We included randomised clinical trials and prospective cohort studies where the population was adults following aSAH, the intervention or exposure was any pharmacological or non-pharmacological therapy, the control group was not restricted and where the incidence of cerebral arterial vasospasm (as defined in the included studies) was reported. We excluded studies with a sample size of < 40 participants due to the potential for bias [13] and resource constraints.

### Search strategy

The search strategy was devised with the assistance of a research librarian. Our primary electronic search was conducted using Medline and EMBASE via the OVID interface and PubMed. We included search terms for subarachnoid haemorrhage, vasospasm, delayed cerebral ischaemia along with specific terms to identify randomised clinical trials and prospective cohort studies. We limited the searches to studies published in English [14] with human subjects. To obtain a contemporary cohort of studies, we limited the search to studies published after 2008. We manually searched the reference lists of review articles. We included published manuscripts and abstracts of conference proceedings. The full search strategy is included in the Supplementary Material.

### Selection process

The titles and abstracts of all identified study reports were independently screened by 2 authors, with reports identified by either reviewer that could potentially meet inclusion criteria retrieved for review as full-text manuscripts. Full-text manuscripts were independently reviewed by 2 authors to assess whether they met all inclusion criteria, with disputes resolved by discussion or resort to a third reviewer.

### Data collection process

Data were extracted onto a specifically designed data extraction form by 2 authors working in teams, with disputes resolved by discussion.

### Data items

Data were collected regarding the details of the included studies (including first author, year of publication, number of study sites, sample size). We collected data regarding the included population (mean age, proportion of female participants), the proportion of participants that had poor neurological state at enrolment, defined as a Hunt and Hess grade of 3, 4 or 5 [15] or a World Federation of Neurological Surgeons clinical grade of 3, 4 or 5 [16], and the proportion of participants with a large subarachnoid blood load, defined as a score of 3 or 4 on the Fisher scale or equivalent [17]. We collected data on the mode of investigation to identify aVSP, whether aVSP was assessed for routinely or only if deemed clinically necessary or, after a screening test determined, it was warranted. For RCTs of interventions designed to reduce the incidence of aVSP, we recorded only the reported incidence of aVSP in the control group for interventions that have been shown to reduce the incidence of aVSP such as clazosentan [18] and cilostazol [19]. We recorded the criteria used to identify cases of aVSP in each of the included studies. Data regarding timing of investigation for aVSP and details of the clinical management protocols were reported infrequently and inconsistently and were unable to be collected in a meaningful fashion. We recorded the proportion of participants diagnosed with DCI only when DCI was defined according to the current standard definition [5].

### Risk of bias assessment

Risk of bias of the included studies was assessed using the ROB V2.0 Cochrane risk of bias tool [20] for randomised clinical trials and the Newcastle–Ottawa score [21] for observational studies. Risk of bias was assessed independently by 2 authors, with disputes resolved by discussion or resort to a third reviewer.

### Effect measures

The primary outcome measure was the incidence of vasospasm. The secondary outcome was the incidence of DCI.

### Data synthesis

Summary statistics are provided to describe the characteristics of the included studies, with counts and proportions for categorical variables and median and interquartile range (IQR) for continuous variables. For the primary outcome, data were pooled using a DerSimonian and Laird random effects model [22] for proportions using the metaprop_one command in STATA, with exact confidence intervals as well as prediction intervals [23]. As a sensitivity analysis, we also pooled the incidence of aVSP using a fixed effect model. We pooled the estimated incidence of aVSP separately for subgroups of studies defined by the mode of investigation used to detect aVSP, whether the radiological assessment of aVSP was performed in all trial participants or only those with clinical, transcranial Doppler or other imaging indications to suggest aVSP, the proportion of trial participants with poor clinical grade (by quartiles) and the proportion of trial participants with poor radiological grade (again by quartiles). We also conducted an analysis stratified by year of publication that was not pre-specified. We pooled the reported incidence of DCI using a random effects model. Within-group heterogeneity was estimated using the *I*^2^ statistic [24]. Between-group heterogeneity was assessed by fitting a single covariate logistic regression model for each specified subgroup. Analyses were performed using STATA (MP version 16.1, College Station, TX, USA).

## Results

### Study selection

**Fig. 1:**
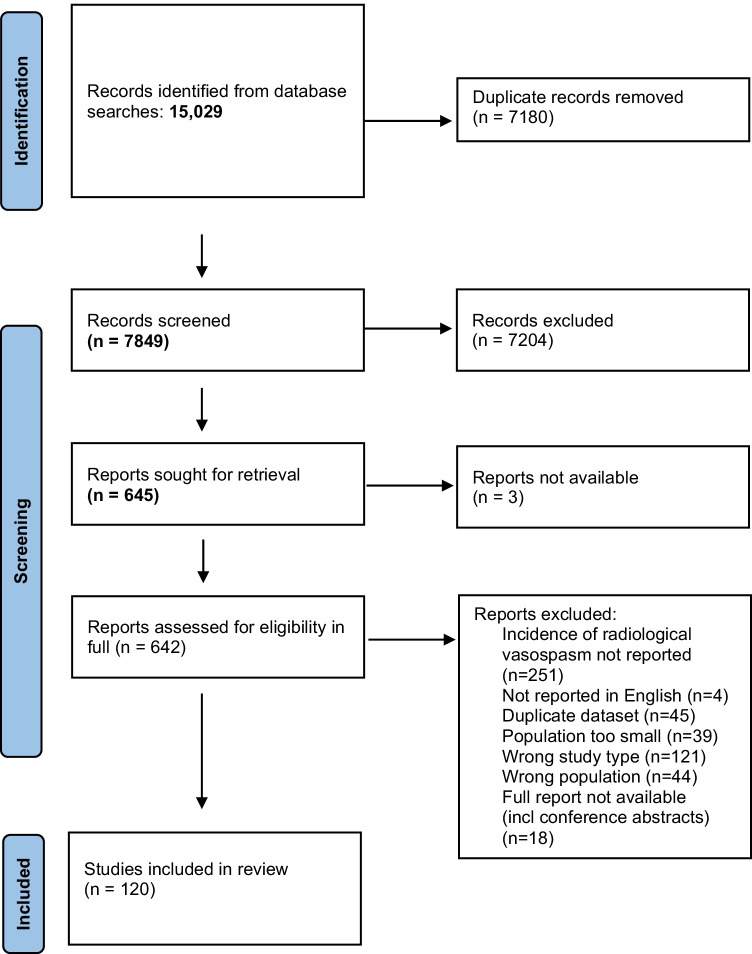
PRISMA flow diagram

The final search was completed on 22 June 2021. There were 15,029 studies identified by the search, with 120 studies included in the meta-analysis, 23 RCTs including 2605 participants and 93 cohort studies including 16,566 participants. The study selection process is shown in Fig. 1, with references to all included studies provided in the Supplement.

The detailed characteristics of the included studies are provided in the Supplement Table S1. In summary, the majority (101/120, 84.2%) of the included studies were single-centre studies; the median number of participants per study was 95 (IQR 58–174). The mean age of the participants in the included trials had a median of 54.7 (IQR 53–57), and the median proportion of trial participants who were female was 0.65 (IQR 0.58 to 0.70). The median proportion with a high-grade CT score was 0.77 (IQR 0.62 to 0.90), and the median proportion with a poor clinical grade at enrolment was 0.43 (IQR 0.31 to 0.55).

### Risk of bias

The risk of bias assessments for the included RCTS are shown in Supplement Fig. S1, and the risk of bias assessments for the included cohort studies are shown in Supplement Table S2. Notably, only 5 of the RCTs were adjudicated as low risk of bias. For the cohort studies, the median score on the Newcastle–Ottawa scale was 7 (IQR 6–8).

### Definition of cerebral arterial vasospasm

The criteria used to define cases of aVSP were variable. The most common criterion to identify cases of aVSP using angiography (either DSA or CTA) was angiographic evidence of narrowing of intracranial arteries to any degree which was used in 34 studies. There were 28 studies that used a percentage reduction in vessel calibre with 7 different thresholds used to diagnose aVSP. There were 13 studies that provided no details of diagnostic criteria for aVSP. Within studies that used transcranial Doppler, 28 defined a middle cerebral artery mean velocity of > 120 cm/s to define aVSP, with a further 25 other criteria used. A total of 41 different definitions of aVSP were described, with 11 studies not specifically reporting the diagnostic criteria. A full account of the criteria used to identify cases of aVSP is shown in Table S3.

### Pooled incidence of aVSP following aSAH

**Fig. 2:**
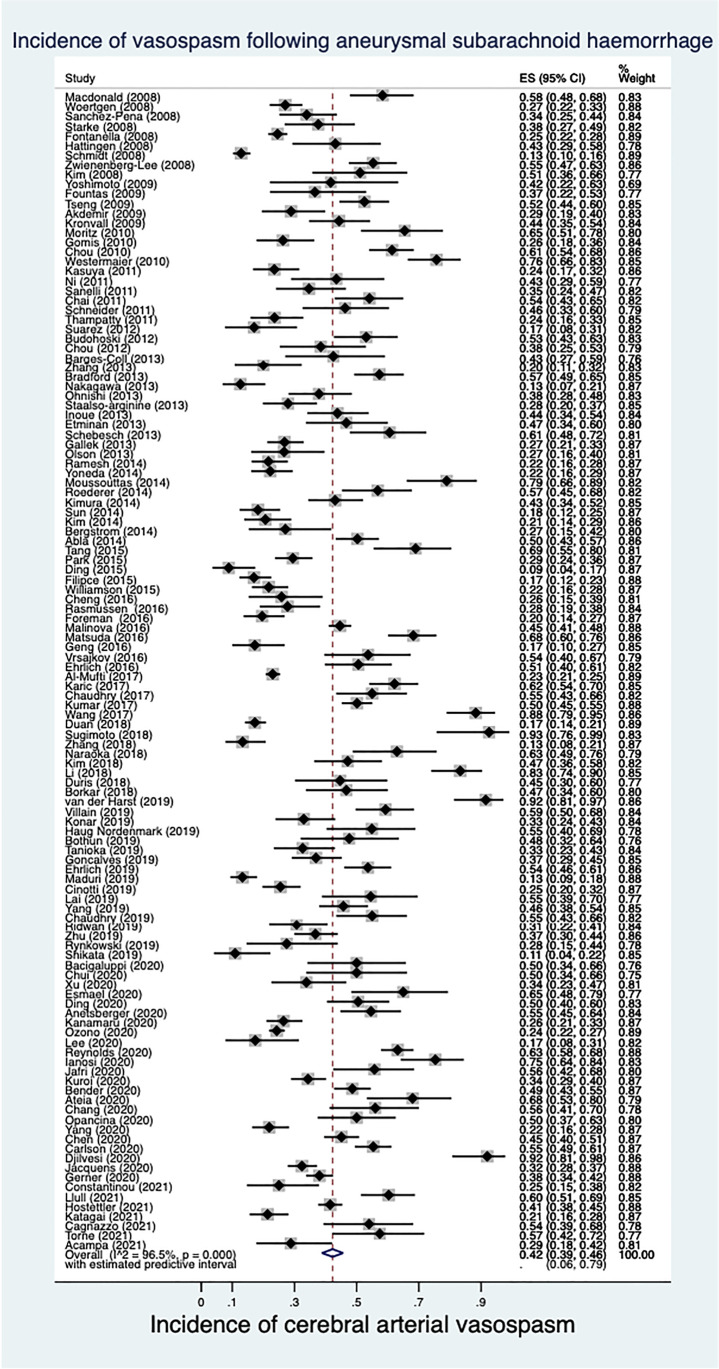
Pooled estimate of the incidence of vasospasm following aneurysmal subarachnoid haemorrhage (random effects model)

The proportion of study participants in whom aVSP was detected in the included studies ranged from 0.09 to 0.93. The pooled estimate of the proportion of study participants who were diagnosed with incident aVSP following aSAH was 0.42 (95% confidence interval (CI) 0.39 to 0.46, *I*^2^ = 96.5%) as shown in Fig. 2 and Table 1. The 95% prediction interval for future studies ranged from 0.06 to 0.79. The pooled estimate of the proportion of study participants who were diagnosed with incident aVSP following aSAH using a fixed effect model was 0.35 (95% CI 0.34 to 0.36) shown in Fig. S2.

**Table 1:** The pooled estimate of the proportion of study participants diagnosed with incident cerebral arterial vasospasm following aneurysmal subarachnoid haemorrhage

	Studies	Participants	Vasospasm incidence	95% CI	*I*^2^	Between-group heterogeneity
Primary outcome	120	19,171	0.42	0.39 to 0.46	96.5%	
Subgroups						
Study type						
RCT	23	2605	0.44	0.35 to 0.53	96.0%	0.63
Cohort	97	16,566	0.42	0.38 to 0.45	96.6%	
Indication for investigation						
Routine	76	11,047	0.47	0.43 to 0.52	96.5%	< 0.005
Symptoms/TCD	22	3550	0.30	0.25 to 0.36	94.0%	
Mode of investigation						
DSA	36	4107	0.39	0.33 to 0.45	94.1%	0.36
CTA	10	1983	0.52	0.34 to 0.70	98.5%	
TCD	31	4731	0.45	0.38 to 0.52	96.5%	
Multiple modes	37	7186	0.43	0.38 to 0.49	96.5%	
Routine screening using DSA	19	2087	0.47	0.39 to 0.56	94.1%	
Proportion of cohort with high grade CT scan (quartiles)						
1	21	3838	0.40	0.32 to 0.48	96.9%	0.84
2	20	2904	0.45	0.37 to 0.53	95.3%	
3	21	4265	0.44	0.35 to 0.53	97.6%	
4	20	2545	0.43	0.39 to 0.53	94.7%	
Proportion of cohort with poor clinical grade (quartiles)						
1	24	3900	0.40	0.32 to 0.48	96.7%	0.59
2	23	3315	0.44	0.37 to 0.52	95.2%	
3	24	4510	0.38	0.33 to 0.44	94.4%	
4	23	3434	0.44	0.38 to 0.54	97.6%	

*CI* confidence interval, *RCT* randomised clinical trial, *TCD* transcranial Doppler, *DSA* digital subtraction angiography, *CTA* computer tomographic angiography, *CT* computed tomography

### Pooled incidence of aVSP in predefined sub-groups

The results of subgroup analyses are shown in Table 1. The pooled estimate for the proportion of study participants in whom aVSP was detected, and the within-subgroup estimates of heterogeneity were similar in RCTs and cohort studies (Fig. S3; Table 1), also in studies grouped by mode of investigation (Fig. S4; Table 1). There was evidence of significant heterogeneity between groups defined by the indication for imaging (Fig. S5; Table 1), with the estimate of the proportion of study participants diagnosed with aVSP in studies where investigation for aVSP was performed routinely (estimated incidence 0.47, 95% CI 0.43 to 0.52, *I*^2^ = 96.5%) compared to studies where investigation for aVSP was only performed when indicated by symptoms or transcranial Doppler (TCD) results (estimated incidence 0.30, 95% CI 0.25 to 0.36, *I*^2^ = 94.0%, *p* for interaction < 0.0005). There were 19 studies including 2087 participants that performed DSAs routinely, and in this subgroup, the pooled estimate of the proportion of study participants diagnosed with aVSP was 0.47 (95% CI 0.39 to 0.56, *I*^2^ = 94.1%) as shown in Table 1 and Fig. S6. There was no evidence of a differential estimate of the proportion of study participants who were diagnosed with aVSP in studies with higher proportions of study participants with high grade CT scans (Table 1; Fig. S7) nor in studies with a greater proportion of study participants with poor clinical grade at presentation (Table 1; Fig. S8). The estimated incidence of aVSP did not vary by year of publication (*p* for between-group heterogeneity = 0.39), with no reduction in the number of studies reporting aVSP as an outcome, nor in the within-group heterogeneity over time as shown in Table S4 and Fig. S9.

### Delayed cerebral ischaemia

There were 26 studies including 4767 participants that reported an incidence of DCI according to our prespecified definition. The pooled estimate of the incidence of DCI was 0.22 (95% CI 0.19 to 0.26, *I*^2^ = 89.6%), as shown in Fig. S10.

## Discussion

We performed a systematic review and meta-analysis to estimate the incidence of aVSP following aSAH and to better understand the causes for variation in the reported incidence. The main finding of this review is that the reported incidence of aVSP following aSAH varies widely. Given that we reported incidence as proportion of the included cohort, it was not surprising that the reported incidence was higher in cohorts where screening for aVSP occurred routinely, compared to those where testing was only performed following a clinical indication or an abnormal TCD screening test. Variability in the reported incidence was not related to the mode of testing performed, nor was it related to the proportion of study participants with high grade CT scans or poor clinical grade. The incidence of aVSP is commonly reported as an outcome in recent studies of aSAH, with no reduction the between study variability in recent years. We identified more than 40 different sets of diagnostic criteria used to define aVSP in these studies.

The association between aVSP and clinical outcome has been questioned [6]. Yet surveys of practice suggest that screening for aVSP and endovascular management of large vessel spasm remain mainstays of practice in the majority of neurocritical care institutions [25], and the management of aVSP remains a focus of contemporary clinical research [26].

The evidence regarding the clinical significance of aVSP remains conflicting. For instance, while the CONSCIOUS-1 trial demonstrated that the endothelin receptor-A antagonist clazosentan prevented angiographic vasospasm but did not reduce mortality or improve outcome [27], a subsequent post hoc analysis which involved blinded re-classification of catheter angiography results suggested a strong correlation between angiographic severity and risk of ischemia [28]. Similarly, in a study that pooled the results of 14 RCTs of pharmaceutical treatment of vasospasm and found that despite a reduction in vasospasm incidence, there was no significant effect on outcome [29]. By contrast, a pooled analysis of 10 RCTs of calcium antagonists in aSAH reported a significant improvement of both death and dependency and aVSP on angiography [30]. Given that these studies and each of the included study within these meta-analyses defined vasospasm differently, the interpretation of these conflicting result is difficult. Without an understanding of the true baseline incidence of aVSP by an agreed standard definition, it is difficult to draw conclusions regarding its association and treatment with outcome or to define degrees of vasospasm severity on highly sensitive modalities like DSA. The Neurocritical Care Society’s Multidisciplinary Consensus guidelines (2011) expressly acknowledge the challenge created by this inconsistency [9]. In contrast, there exists a standard definition for DCI developed by consensus in 2010 [5] allowing a consistent estimate of its impact on outcome [31] and increasingly detailed exploration of its pathogenesis [32]. The unruptured intracranial aneurysms and SAH Common Data Elements project has provided a template for data elements to be reported with respect to vascular imaging [33] after subarachnoid haemorrhage, without providing a specific criteria to diagnose or exclude aVSP in this context.

This study reports for the first time a robust estimate of the incidence of aVSP and demonstrates clearly the impact of differing modalities and clinical contexts on this baseline incidence. By examining all modes of investigation and deriving a pooled estimate of incidence, this study adds to the recent analysis by Darsaut et al. which identified heterogenous definitions of cerebral vasospasm defined by DSA alone, as well as poor inter-observer agreement on DSA interpretation [34].

### Strengths and limitations

There are a number of strengths to this systematic review. This study was undertaken according to a pre-specified protocol and is reported according to current best practice standards [12]. The broad inclusion criteria ensure the generalizability of the results. It provides a contemporary overview of the current diagnostic standards in the reported literature. However, there are certain limitations of this study. Although we attempted to contact study authors to obtain additional information not provided in published reports of studies, this was not possible for all studies. Also, there may be other explanations for the variation in the reported incidence of aVSP that are not reported at a study level that may explain some of the heterogeneity, with patient level data being required to determine the role of such factors.

### Implications for clinical practice and for future research

This study has important implications for clinicians as well as researchers. When interpreting the literature regarding the role that aVSP plays in delayed neurological deterioration following aSAH, clinicians should play close attention to the methods used to detect aVSP and to the definition of aVSP. It seems premature to draw conclusions about the potential effectiveness or lack of effectiveness of interventions to prevent or treat aVSP prior to having a common understanding of the condition. Furthermore, when aVSP is reported on angiography, standardised criteria for severity could be used to allow higher fidelity stratification of patients who do have aVSP. Similarly, for researchers, it is essential that a standard definition and diagnostic criteria for aVSP are established in order to better understand the role that aVSP may play in the pathophysiology of delayed neurological injury following aSAH.

## Conclusion

The incidence of cerebral arterial vasospasm following aSAH varies widely from 9 to 93% of study participants. Heterogeneity in the reported incidence may be due to variation in the criteria used to diagnose aVSP. A standard set of diagnostic criteria for reporting aVSP following aSAH is necessary to resolve the role that aVSP plays in delayed neurological deterioration in patients with aSAH.

## Supplementary Information

Below is the link to the electronic supplementary material.

Supplementary file1 (DOCX 21 KB)

Supplementary file2 (DOCX 5341 KB)

## Data Availability

Extracted study data available by request to authors. Search strategy included in Supplementary Material.

## Code availability

Meta-analytic code (Stata, College Station, TX: Stata Press. StataCorp. 2019) available by request to authors.

## Declarations

### Reporting standards

The completed PRIMSA Checklist has been included as a supplement. This manuscript has not been published elsewhere and is not under consideration by another journal.
