# Supplementary material for: The incidence of cerebral arterial vasospasm following aneurysmal subarachnoid haemorrhage: a systematic review and meta-analysis
Source: Neuroradiology. 2022 Jul 7;64(12):2381–9. doi: 10.1007/s00234-022-03004-w (PMC9643195; doi:10.1007/s00234-022-03004-w)
Supplement: Supplementary file 2 — Supplementary file2 (DOCX 5341 KB) [file 234_2022_3004_MOESM2_ESM.docx]

**Supplementary Material**

**Table of Contents**

[Full List of Included Studies 2](#_Toc106693190)

[Table S1: Characteristics of the included studies 9](#_Toc106693191)

[Figure S1: Risk of Bias assessment for included Randomised Controlled Trials (Cochrane ROB2 tool) 14](#_Toc106693192)

[Table S2: Risk of Bias assessment for included prospective cohort studies (Newcastle-Ottawa Scale) 15](#_Toc106693193)

[Table S3: Criteria used to define arterial vasospasm in the included studies 18](#_Toc106693194)

[Table S4: Estimated pooled incidence of cerebral arterial vasospasm following aneurysmal subarachnoid haemorrhage by year of publication. 20](#_Toc106693195)

[Figure S2: The pooled estimated incidence of cerebral arterial vasospasm following aneurysmal subarachnoid haemorrhage: fixed effect model (sensitivity analysis) 21](#_Toc106693196)

[Figure S3: The pooled estimated incidence of cerebral arterial vasospasm following aneurysmal subarachnoid haemorrhage: by study type (random effects model) 22](#_Toc106693197)

[Figure S4: The pooled estimated incidence of cerebral arterial vasospasm following aneurysmal subarachnoid haemorrhage: by mode of investigation (random effects model) 23](#_Toc106693198)

[Figure S5: The pooled estimated incidence of cerebral arterial vasospasm following aneurysmal subarachnoid haemorrhage: indication for imaging (random effects model) 24](#_Toc106693199)

[Figure S6: The pooled estimated incidence of cerebral arterial vasospasm following aneurysmal subarachnoid haemorrhage in studies performing routine DSA (random effects model) 25](#_Toc106693200)

[Figure S7: The pooled estimated incidence of cerebral arterial vasospasm following aneurysmal subarachnoid haemorrhage: by proportion of study participants with high grade CT scans (random effects model) 26](#_Toc106693201)

[Figure S8: The pooled estimated incidence of cerebral arterial vasospasm following aneurysmal subarachnoid haemorrhage: by proportion of study participants with poor clinical grade at presentation (random effects model) 27](#_Toc106693202)

[Figure S9: The pooled estimated incidence of cerebral arterial vasospasm by year (random effects model) 28](#_Toc106693203)

[Figure S10: The pooled estimated incidence of delayed cerebral ischaemia following aneurysmal subarachnoid haemorrhage (random effects model) 28](#_Toc106693204)

[Search Strategy 30](#_Toc106693205)

Full List of Included Studies

1. Abla AA, Wilson DA, Williamson RW, Nakaji P, McDougall CG, Zabramski JM, et al. The relationship between ruptured aneurysm location, subarachnoid hemorrhage clot thickness, and incidence of radiographic or symptomatic vasospasm in patients enrolled in a prospective randomized controlled trial. Journal of neurosurgery. 2014;120(2):391-7.

2. Acampa M, Bongiorno M, Lazzerini PE, Catania C, Domenichelli C, Guideri F, et al. Increased Arterial Stiffness is a Predictor of Delayed Ischaemic Stroke After Subarachnoid Haemorrhage. Heart Lung and Circulation. 2021;30(4):525-30.

3. Akdemir H, Kulakszoglu EO, Tucer B, Menku A, Postalc L, Gunald O. Magnesium sulfate therapy for cerebral vasospasm after aneurysmal subarachnoid hemorrhage. Neurosurgery Quarterly. 2009;19(1):35-9.

4. Al-Mufti F, Roh D, Lahiri S, Meyers E, Witsch J, Frey HP, et al. Ultra-early angiographic vasospasm associated with delayed cerebral ischemia and infarction following aneurysmal subarachnoid hemorrhage. Journal of Neurosurgery. 2017;126(5):1545-51.

5. Anetsberger A, Gempt J, Blobner M, Ringel F, Bogdanski R, Heim M, et al. Impact of Goal-Directed Therapy on Delayed Ischemia After Aneurysmal Subarachnoid Hemorrhage: Randomized Controlled Trial. Stroke. 2020;51(8):2287-96.

6. Ateia AM, Elbassiouny A, El-Nabi SH, Fahmy NA, Ibrahim MH, El-Garawani I, et al. Predictive value of haptoglobin genotype as a risk of cerebral vasospasm after aneurysmal subarachnoid hemorrhage. Clinical Neurology and Neurosurgery. 2020;199(no pagination).

7. Bacigaluppi S, Ivaldi F, Bragazzi NL, Benvenuto F, Gallo F, D'Andrea A, et al. An Early Increase of Blood Leukocyte Subsets in Aneurysmal Subarachnoid Hemorrhage Is Predictive of Vasospasm. Frontiers in Neurology. 2020;11(no pagination).

8. Barges-Coll J, Perez-Neri I, Avendano J, Mendez-Rosito D, Gomez-Amador JL, Rios C. Plasma taurine as a predictor of poor outcome in patients with mild neurological deficits after aneurysmal subarachnoid hemorrhage. Journal of Neurosurgery. 2013;119(4):1021-7.

9. Bender M, Richter E, Schwarm FP, Kolodziej MA, Uhl E, Reinges MHT, et al. Transcranial Doppler Sonography Defined Vasospasm, Ischemic Brain Lesions, and Delayed Ischemic Neurological Deficit in Younger and Elderly Patients after Aneurysmal Subarachnoid Hemorrhage. World Neurosurgery. 2020;138:e718-e24.

10. Bergstrom A, Staalso JM, Romner B, Olsen NV. Impaired endothelial function after aneurysmal subarachnoid haemorrhage correlates with arginine:asymmetric dimethylarginine ratio. British Journal of Anaesthesia. 2014;112(2):311-8.

11. Borkar SA, Singh M, Kale SS, Suri A, Chandra PS, Kumar R, et al. Spinal cerebrospinal fluid drainage for prevention of vasospasm in aneurysmal subarachnoid hemorrhage: A prospective, randomized controlled study. Asian journal of neurosurgery. 2018;13(2):238.

12. Bothun ML, Haaland OA, Moen G, Logallo N, Svendsen F, Thomassen L, et al. Impaired cerebrovascular reactivity may predict delayed cerebral ischemia after aneurysmal subarachnoid hemorrhage. Journal of the Neurological Sciences. 2019;407(no pagination).

13. Bradford CM, Finfer S, O'Connor A, Yarad E, Firth R, McCallister R, et al. A randomised controlled trial of induced hypermagnesaemia following aneurysmal subarachnoid haemorrhage. Critical care and resuscitation : journal of the Australasian Academy of Critical Care Medicine. 2013;15(2):119-25.

14. Budohoski KP, Czosnyka M, Smielewski P, Kasprowicz M, Helmy A, Bulters D, et al. Impairment of cerebral autoregulation predicts delayed cerebral ischemia after subarachnoid hemorrhage: A prospective observational study. Stroke. 2012;43(12):3230-7.

15. Cagnazzo F, Chalard K, Lefevre PH, Garnier O, Derraz I, Dargazanli C, et al. Optimal intracranial pressure in patients with aneurysmal subarachnoid hemorrhage treated with coiling and requiring external ventricular drainage. Neurosurgical Review. 2021;44(2):1191-204.

16. Carlson AP, Hanggi D, Wong GK, Etminan N, Mayer SA, Aldrich F, et al. Single-Dose Intraventricular Nimodipine Microparticles Versus Oral Nimodipine for Aneurysmal Subarachnoid Hemorrhage. Stroke. 2020;51(4):1142-9.

17. Chai WN, Sun XC, Lv FJ, Wan B, Jiang L. Clinical study of changes of cerebral microcirculation in cerebral vasospasm after SAH. Early Brain Injury or Cerebral Vasospasm: Volume 1: Pathophysiology. 2011;Acta Neurochirurgica, Supplementum.(110 1):225-8.

18. Chang JJ, Triano M, Corbin MJ, Desale S, Liu AH, Felbaum DR, et al. Transcranial Doppler velocity and associations with delayed cerebral ischemia in aneurysmal subarachnoid Hemorrhage. Journal of the Neurological Sciences. 2020;415:116934.

19. Chaudhry SR, Frede S, Seifert G, Kinfe TM, Niemela M, Lamprecht A, et al. Temporal profile of serum mitochondrial DNA (mtDNA) in patients with aneurysmal subarachnoid hemorrhage (aSAH). Mitochondrion. 2019;47:218-26.

20. Chaudhry SR, Stoffel-Wagner B, Kinfe TM, Guresir E, Vatter H, Dietrich D, et al. Elevated Systemic IL-6 Levels in Patients with Aneurysmal Subarachnoid Hemorrhage Is an Unspecific Marker for Post-SAH Complications. International journal of molecular sciences. 2017;18(12).

21. Chen J, Li M, Zhu X, Chen L, Yang S, Zhang C, et al. Atorvastatin reduces cerebral vasospasm and infarction after aneurysmal subarachnoid hemorrhage in elderly Chinese adults. Aging (Albany NY). 2020;12(3):2939-51.

22. Cheng XQ, Chen Q, Zhou CS, Li JR, Zhang ZJ, Zhang LJ, et al. Whole-brain CT perfusion combined with CT angiography for ischemic complications following microsurgical clipping and endovascular coiling of ruptured intracranial aneurysms. J Clin Neurosci. 2016;26:50-6.

23. Chou CH, Reed SD, Allsbrook JS, Steele JL, Schulman KA, Alexander MJ. Costs of vasospasm in patients with aneurysmal subarachnoid hemorrhage. Neurosurgery. 2010;67(2):345-51.

24. Chou SHY, Feske SK, Atherton J, Konigsberg RG, De Jager PL, Du R, et al. Early elevation of serum tumor necrosis factor-alpha is associated with poor outcome in subarachnoid hemorrhage. Journal of Investigative Medicine. 2012;60(7):1054-8.

25. Chui J, Craen R, Dy-Valdez C, Alamri R, Boulton M, Pandey S, et al. Early goal-directed Therapy during Endovascular Coiling Procedures following Aneurysmal Subarachnoid Hemorrhage: A Pilot Prospective Randomized Controlled Study. Journal of Neurosurgical Anesthesiology. 2020.

26. Cinotti R, Putegnat JB, Lakhal K, Desal H, Chenet A, Buffenoir K, et al. Evolution of neurological recovery during the first year after subarachnoid haemorrhage in a French university centre. Anaesth Crit Care Pain Med. 2019;38(3):251-7.

27. Constantinou S, Cheng PH, Holmes D, McGarrity P, Abouharb A, Simms HN. Prevention of hyponatraemia with prophylactic oral sodium chloride in good grade aneurysmal subarachnoid haemorrhage. Interdisciplinary Neurosurgery: Advanced Techniques and Case Management. 2021;25(no pagination).

28. Ding CY, Cai HP, Ge HL, Yu LH, Lin YX, Kang DZ. Is Admission Lipoprotein-Associated Phospholipase A2 a Novel Predictor of Vasospasm and Outcome in Patients With Aneurysmal Subarachnoid Hemorrhage? Neurosurgery. 2020;86(1):122-31.

29. Ding W, Gao N, Li MX, Ding LJ, Li FF, Mou L. Clinical evaluation of the efficacy of the combination of aneurysm embolization and cerebrospinal fluid replacement in the treatment of aneurysmal subarachnoid hemorrhage. European Review for Medical and Pharmacological Sciences. 2015;19(3):402-5.

30. Djilvesi D, Horvat I, Jelaca B, Golubovic J, Pajicic F, Vulekovic P. Comparison of radiological versus clinical cerebral vasospasm after aneurysmal subarachnoid hemorrhage: is vasospasm always present? Neurological Research. 2020;42(12):1027-33.

31. Duan W, Pan Y, Wang C, Zhao X, Wang Y, Liu L. Risk Factors and Clinical Impact of Delayed Cerebral Ischemia after Aneurysmal Subarachnoid Hemorrhage: Analysis from the China National Stroke Registry. Neuroepidemiology. 2018;50(3-4):128-36.

32. Duris K, Neuman E, Vybihal V, Juraa V, Gottwaldova J, Kyr M, et al. Early Dynamics of Interleukin-6 in Cerebrospinal Fluid after Aneurysmal Subarachnoid Hemorrhage. Journal of Neurological Surgery, Part A: Central European Neurosurgery. 2018;79(2):145-51.

33. Ehrlich G, Kirschning T, Wenz H, Hegewald AA, Groden C, Schmiedek P, et al. Is There an Influence of Routine Daily Transcranial Doppler Examination on Clinical Outcome in Patients after Aneurysmal Subarachnoid Hemorrhage? World Neurosurgery. 2016;88:214-21.

34. Ehrlich G, Kirschning T, Wenz H, Hegewald AA, Neumaier-Probst E, Seiz-Rosenhagen M. Outcome of Oral and Intra-arterial Nimodipine Administration after Aneurysmal Subarachnoid Haemorrhage - A Single-centre Study. In Vivo. 2019;33(6):1967-75.

35. Esmael A, Belal T, Eltoukhy K. Transcranial doppler for early prediction of cognitive impairment after aneurysmal subarachnoid hemorrhage and the associated clinical biomarkers. Stroke Research and Treatment. 2020(pagination).

36. Etminan N, Beseoglu K, Heiroth HJ, Turowski B, Steiger HJ, Hanggi D. Early perfusion computerized tomography imaging as a radiographic surrogate for delayed cerebral ischemia and functional outcome after subarachnoid hemorrhage. Stroke. 2013;44(5):1260-6.

37. Filipce V, Caparoski A. The Effects of Vasospasm and Re-Bleeding on the Outcome of Patients with Subarachnoid Hemorrhage from Ruptured Intracranial Aneurysm. Prilozi Makedonska Akademija Na Naukite I Umetnostite Oddelenie Za Medicinski Nauki. 2015;36(3):77-82.

38. Fontanella M, Valfre W, Benech F, Carlino C, Garbossa D, Ferrio M, et al. Vasospasm after SAH due to aneurysm rupture of the anterior circle of Willis: Value of TCD monitoring. Neurological Research. 2008;30(3):256-61.

39. Foreman PM, Chua M, Harrigan MR, Fisher WS, Vyas NA, Lipsky RH, et al. Nosocomial infections are associated with delayed cerebral ischemia in aneurysmal subarachnoid hemorrhage. Stroke Conference: American Heart Association/American Stroke Association. 2016;47(SUPPL. 1).

40. Fountas KN, Tasiou A, Kapsalaki EZ, Paterakis KN, Grigorian AA, Lee GP, et al. Serum and cerebrospinal fluid C-reactive protein levels as predictors of vasospasm in aneurysmal subarachnoid hemorrhage. Clinical article. Neurosurgical focus. 2009;26(5):E22.

41. Gallek M, Alexander S, Crago E, Sherwood P, Horowitz M, Poloyac S, et al. Endothelin-1 and endothelin receptor gene variants and their association with negative outcomes following aneurysmal subarachnoid hemorrhage. Biological research for nursing. 2013;15(4):390-7.

42. Geng L, Ma F, Liu Y, Mu Y, Zou Z. Massive Cerebrospinal Fluid Replacement Reduces Delayed Cerebral Vasospasm After Embolization of Aneurysmal Subarachnoid Hemorrhage. Medical science monitor : international medical journal of experimental and clinical research. 2016;22:2404-8.

43. Gerner ST, Reichl J, Custal C, Brandner S, Eyupoglu IY, Lucking H, et al. Long-Term complications and influence on outcome in patients surviving spontaneous subarachnoid hemorrhage. Cerebrovascular Diseases. 2020;49(3):307-15.

44. Gomis P, Graftieaux JP, Sercombe R, Hettler D, Scherpereel B, Rousseaux P. Randomized, double-blind, placebo-controlled, pilot trial of high-dose methylprednisolone in aneurysmal subarachnoid hemorrhage. Journal of neurosurgery. 2010;112(3):681-8.

45. Goncalves B, Kurtz P, Turon R, Santos T, Prazeres M, Righy C, et al. Incidence and impact of sepsis on long-term outcomes after subarachnoid hemorrhage: a prospective observational study. Annals of Intensive Care. 2019;9(1).

46. Hattingen E, Blasel S, Dettmann E, Vatter H, Pilatus U, Seifert V, et al. Perfusion-weighted MRI to evaluate cerebral autoregulation in aneurysmal subarachnoid haemorrhage. Neuroradiology. 2008;50(11):929-38.

47. Haug Nordenmark T, Karic T, Sorteberg W, Sorteberg A. Predictors of cognitive function in the acute phase after aneurysmal subarachnoid hemorrhage. Acta Neurochirurgica. 2019;161(1):177-84.

48. Hostettler IC, Sebok M, Ambler G, Muroi C, Prommel P, Neidert MC, et al. Validation and optimization of barrow neurological institute score in prediction of adverse events and functional outcome after subarachnoid hemorrhage-creation of the HATCH (Hemorrhage, age, treatment, clinical state, hydrocephalus) score. Neurosurgery. 2021;88(1):96-105.

49. Ianosi B, Rass V, Gaasch M, Huber L, Lindner A, Hackl WO, et al. An Observational Study on the Use of Intravenous Non-Opioid Analgesics and Antipyretics in Poor-Grade Subarachnoid Hemorrhage: Effects on Hemodynamics and Systemic and Brain Temperature. Ther Hypothermia Temp Manag. 2020;10(1):27-36.

50. Inoue M, Sasaki T, Takazawa H, Morita T, Narisawa A, Saito A, et al. Symptomatic vasospasm in elderly patients with aneurysmal subarachnoid hemorrhage: Comparison with nonelderly patients. Cerebral Vasospasm: Neurovascular Events After Subarachnoid Hemorrhage. 2013;Acta Neurochirurgica, Supplementum. 115:281-4.

51. Jacquens A, Shotar E, Bombled C, Glemain B, Sourour NA, Nouet A, et al. Is Anatomical Variations a Risk Factor for Cerebral Vasospasm in Anterior Communicating Complex Aneurysms Rupture? Stroke. 2020;51(3):998-1001.

52. Jafri H, Diringer MN, Allen M, Zazulia AR, Zipfel GJ, Dhar R. Burden of cerebral hypoperfusion in patients with delayed cerebral ischemia after subarachnoid hemorrhage. Journal of Neurosurgery. 2020;132(6):1872-9.

53. Kanamaru H, Kawakita F, Asada R, Miura Y, Shiba M, Toma N, et al. Prognostic factors varying with age in patients with aneurysmal subarachnoid hemorrhage. J Clin Neurosci. 2020;76(pp 118-125).

54. Karic T, Roe C, Nordenmark TH, Becker F, Sorteberg W, Sorteberg A. Effect of early mobilization and rehabilitation on complications in aneurysmal subarachnoid hemorrhage. Journal of Neurosurgery. 2017;126(2):518-26.

55. Kasuya H. Clinical trial of nicardipine prolonged-release implants for preventing cerebral vasospasm: Multicenter cooperative study in Tokyo. Early Brain Injury or Cerebral Vasospasm: Volume 2: Clinical Management. 2011;Acta Neurochirurgica, Supplementum.(110 2):165-7.

56. Katagai T, Naraoka M, Shimamura N, Asano K, Ohkuma H. Effect of Surgical Arachnoid Plasty on Functional Outcome in Aneurysmal Subarachnoid Hemorrhage. World Neurosurgery. 2021;147(pp e373-e381).

57. Kim BJ, Kim Y, Kim SE, Jeon JP. Study of Correlation Between Hp alpha1 Expression of Haptoglobin 2-1 and Clinical Course in Aneurysmal Subarachnoid Hemorrhage. World Neurosurg. 2018;117:e221-e7.

58. Kim GH, Kellner CP, Hahn DK, Desantis BM, Musabbir M, Starke RM, et al. Monocyte chemoattractant protein-1 predicts outcome and vasospasm following aneurysmal subarachnoid hemorrhage. Journal of neurosurgery. 2008;109(1):38-43.

59. Kim JH, Yi HJ, Ko Y, Kim YS, Kim DW, Kim JM. Effectiveness of papaverine cisternal irrigation for cerebral vasospasm after aneurysmal subarachnoid hemorrhage and measurement of biomarkers. Neurological Sciences. 2014;35(5):715-22.

60. Kimura H, Okamura Y, Chiba Y, Shigeru M, Ishii T, Hori T, et al. Cilostazol administration with combination enteral and parenteral nutrition therapy remarkably improves outcome after subarachnoid hemorrhage. Acta Neurochirurgica, Supplementum. 2014;120:147-52.

61. Konar SK, Ramesh S, Christopher R, Prasanthi A, Bhat DI, Shukla D, et al. The Correlation of Endothelial Nitric Oxide Synthase (eNOS) Polymorphism and Other Risk Factors with Aneurysmal Subarachnoid Hemorrhage: A Case-Control Study. Neurol India. 2019;67(4):1006-12.

62. Kronvall E, Undren P, Romner B, Saveland H, Cronqvist M, Nilsson OG. Nimodipine in aneurysmal subarachnoid hemorrhage: A randomized study of intravenous or peroral administration - Clinical article. Journal of Neurosurgery. 2009;110(1):58-63.

63. Kumar MA, Levine J, Faerber J, Elliott JP, Winn HR, Doerfler S, et al. The Effects of Red Blood Cell Transfusion on Functional Outcome after Aneurysmal Subarachnoid Hemorrhage. World Neurosurg. 2017;108:807-16.

64. Kuroi Y, Ohbuchi H, Arai N, Takahashi Y, Hagiwara S, Sasahara A, et al. Twelve-year single critical care center experience of nicardipine prolonged-release implants in patients with subarachnoid hemorrhage: a propensity score matching analysis. Journal of neurointerventional surgery. 2020;12(8):774-6.

65. Lai PMR, See AP, Silva MA, Gormley WB, Frerichs KU, Aziz-Sultan MA, et al. Noninfectious Fever in Aneurysmal Subarachnoid Hemorrhage: Association with Cerebral Vasospasm and Clinical Outcome. World Neurosurgery. 2019;122(pp e1014-e1019).

66. Lee DH, Cho SY, Yang SB, Lee HM, Shin HS, Lee SH, et al. Efficacy of Acupuncture Treatment to Prevent Cerebral Vasospasm after Subarachnoid Hemorrhage: A Double-Blind, Randomized Placebo-Controlled Trial. Journal of Alternative and Complementary Medicine. 2020;26(12):1182-9.

67. Li DD, Chang JY, Zhou CX, Cui JB. Clinical diagnosis of cerebral vasospasm after subarachnoid hemorrhage by using transcranial Doppler sonography. European Review for Medical and Pharmacological Sciences. 2018;22(7):2029-35.

68. Llull L, Maya G, Torne R, Mellado-Artigas R, Renu A, Lopez-Rueda A, et al. Stroke units could be a valid alternative to intensive care units for patients with low-grade aneurysmal subarachnoid haemorrhage. European Journal of Neurology. 2021;28(2):500-8.

69. Macdonald RL, Kassell NF, Mayer S, Ruefenacht D, Schmiedek P, Weidauer S, et al. Clazosentan to overcome neurological ischemia and infarction occurring after subarachnoid hemorrhage (CONSCIOUS-1): randomized, double-blind, placebo-controlled phase 2 dose-finding trial. Stroke. 2008;39(11):3015-21.

70. Maduri R, Starnoni D, Rocca A, Bervini D, Zumofen DW, Stienen MN, et al. Ruptured posterior circulation aneurysms: epidemiology, patterns of care, and outcomes from the Swiss SOS national registry. Acta Neurochirurgica. 2019;161(4):769-79.

71. Malinova V, Schatlo B, Voit M, Suntheim P, Rohde V, Mielke D. Identification of specific age groups with a high risk for developing cerebral vasospasm after aneurysmal subarachnoid hemorrhage. Neurosurg Rev. 2016;39(3):429-36.

72. Matsuda N, Naraoka M, Ohkuma H, Shimamura N, Ito K, Asano K, et al. Effect of Cilostazol on Cerebral Vasospasm and Outcome in Patients with Aneurysmal Subarachnoid Hemorrhage: A Randomized, Double-Blind, Placebo-Controlled Trial. Cerebrovascular diseases (Basel, Switzerland). 2016;42(1-2):97-105.

73. Moritz S, Warnat J, Bele S, Graf BM, Woertgen C. The prognostic value of NSE and S100B from serum and cerebrospinal fluid in patients with spontaneous subarachnoid hemorrhage. Journal of Neurosurgical Anesthesiology. 2010;22(1):21-31.

74. Moussouttas M, Lai EW, Huynh TT, James J, Stocks-Dietz C, Dombrowski K, et al. Association between acute sympathetic response, early onset vasospasm, and delayed vasospasm following spontaneous subarachnoid hemorrhage. J Clin Neurosci. 2014;21(2):256-62.

75. Nakagawa I, Hironaka Y, Nishimura F, Takeshima Y, Matsuda R, Yamada S, et al. Early inhibition of natriuresis suppresses symptomatic cerebral vasospasm in patients with aneurysmal subarachnoid hemorrhage. Cerebrovascular Diseases. 2013;35(2):131-7.

76. Naraoka M, Matsuda N, Shimamura N, Asano K, Akasaka K, Takemura A, et al. Long-acting statin for aneurysmal subarachnoid hemorrhage: A randomized, double-blind, placebo-controlled trial. Journal of Cerebral Blood Flow and Metabolism. 2018;38(7):1190-8.

77. Ni W, Gu YX, Song DL, Leng B, Li PL, Mao Y. The relationship between IL-6 in CSF and occurrence of vasospasm after subarachnoid hemorrhage. Acta Neurochirurgica - Supplement. 2011;110(Pt 1):203-8.

78. Ohnishi H, Iihara K, Kaku Y, Yamauchi K, Fukuda K, Nishimura K, et al. Haptoglobin phenotype predicts cerebral vasospasm and clinical deterioration after aneurysmal subarachnoid hemorrhage. Journal of Stroke and Cerebrovascular Diseases. 2013;22(4):520-6.

79. Olson DM, Zomorodi M, Britz GW, Zomorodi AR, Amato A, Graffagnino C. Continuous cerebral spinal fluid drainage associated with complications in patients admitted with subarachnoid hemorrhage. Journal of neurosurgery. 2013;119(4):974-80.

80. Opancina V, Lukic S, Jankovic S, Vojinovic R, Mijailovic M. Risk factors for cerebral vasospasm in patients with aneurysmal subarachnoid hemorrhage. Open Medicine. 2020;15(1):598-604.

81. Ozono I, Ikawa F, Hidaka T, Yoshiyama M, Matsuda S, Michihata N, et al. Risk Factor for Poor Outcome in Elderly Patients with Aneurysmal Subarachnoid Hemorrhage Based on Post Hoc Analysis of the Modified WFNS Scale Study. World Neurosurgery. 2020;141(pp e466-e473).

82. Park S, Yang N, Seo E. The Effectiveness of Lumbar Cerebrospinal Fluid Drainage to Reduce the Cerebral Vasospasm after Surgical Clipping for Aneurysmal Subarachnoid Hemorrhage. Journal of Korean Neurosurgical Society. 2015;57(3):167-73.

83. Ramesh SS, Christopher R, Devi BI, Bhat DI. Evaluation the role of oxidative stress and nitric oxide in cerebral vasospasm following aneurysmal subarachnoid hemorrhage. Indian Journal of Clinical Biochemistry. 2016;31 (1 Supplement 1):S86-S7.

84. Rasmussen R, Stavngaard T, Jessing IR, Skjoth-Rasmussen J, Olsen NV, Ostrowski SR, et al. High plasma levels of neuropeptide y correlate with good clinical outcome but are not correlated to cerebral blood flow or vasospasm after subarachnoid hemorrhage. Journal of Neurosurgical Anesthesiology. 2016;28(1):65-70.

85. Reynolds RA, Amin SN, Jonathan SV, Tang AR, Lan M, Wang C, et al. Hyperoxemia and Cerebral Vasospasm in Aneurysmal Subarachnoid Hemorrhage. Neurocritical Care. 2020.

86. Ridwan S, Zur B, Kurscheid J, Esche J, Kristof R, Klingmuller D, et al. Hyponatremia After Spontaneous Aneurysmal Subarachnoid Hemorrhage-A Prospective Observational Study. World Neurosurgery. 2019;129:e538-e44.

87. Roederer A, Holmes JH, Smith MJ, Lee I, Park S. Prediction of significant vasospasm in aneurysmal subarachnoid hemorrhage using automated data. Neurocrit Care. 2014;21(3):444-50.

88. Rynkowski CB, de Oliveira Manoel AL, Dos Reis MM, Puppo C, Worm PV, Zambonin D, et al. Early Transcranial Doppler Evaluation of Cerebral Autoregulation Independently Predicts Functional Outcome After Aneurysmal Subarachnoid Hemorrhage. Neurocrit Care. 2019;31(2):253-62.

89. Sanchez-Pena P, Pereira AR, Sourour NA, Biondi A, Lejean L, Colonne C, et al. S100B as an additional prognostic marker in subarachnoid aneurysmal hemorrhage. Critical Care Medicine. 2008;36(8):2267-73.

90. Sanelli PC, Ugorec I, Johnson CE, Tan J, Segal AZ, Fink M, et al. Using quantitative CT perfusion for evaluation of delayed cerebral ischemia following aneurysmal subarachnoid hemorrhage. Ajnr: American Journal of Neuroradiology. 2011;32(11):2047-53.

91. Schebesch KM, Brawanski A, Bele S, Schodel P, Herbst A, Brundl E, et al. Neuropeptide Y - An early biomarker for cerebral vasospasm after aneurysmal subarachnoid hemorrhage. Neurological Research. 2013;35(10):1038-43.

92. Schmidt JM, Wartenberg KE, Fernandez A, Claassen J, Rincon F, Ostapkovich ND, et al. Frequency and clinical impact of asymptomatic cerebral infarction due to vasospasm after subarachnoid hemorrhage: Clinical article. Journal of Neurosurgery. 2008;109(6):1052-9.

93. Schneider UC, Dreher S, Hoffmann KT, Schmiedek P, Kasuya H, Vajkoczy P. The use of nicardipine prolonged release implants (NPRI) in microsurgical clipping after aneurysmal subarachnoid haemorrhage: comparison with endovascular treatment. Acta Neurochir (Wien). 2011;153(11):2119-25.

94. Shikata E, Tamura T, Shinno K, Okayama Y, Shinohara N, Shimada K, et al. Importance of Managing the Water-Electrolyte Balance by Delivering the Optimal Minimum Amount of Water and Sodium After Subarachnoid Hemorrhage. World Neurosurgery. 2019;129(pp e352-e360).

95. Staalso JM, Bergstrom A, Edsen T, Weikop P, Romner B, Olsen NV. Low plasma arginine: Asymmetric dimethyl arginine ratios predict mortality after intracranial aneurysm rupture. Stroke. 2013;44(5):1273-81.

96. Starke RM, Kim GH, Komotar RJ, Hickman ZL, Black EM, Rosales MB, et al. Endothelial nitric oxide synthase gene single-nucleotide polymorphism predicts cerebral vasospasm after aneurysmal subarachnoid hemorrhage. Journal of cerebral blood flow and metabolism : official journal of the International Society of Cerebral Blood Flow and Metabolism. 2008;28(6):1204-11.

97. Suarez JI, Martin RH, Calvillo E. Albumin in subarachnoid hemorrhage: The alisah pilot study results. Neurocrit Care. 2011;1):S5.

98. Sugimoto K, Nomura S, Shirao S, Inoue T, Ishihara H, Kawano R, et al. Cilostazol decreases duration of spreading depolarization and spreading ischemia after aneurysmal subarachnoid hemorrhage. Annals of Neurology. 2018;84(6):873-85.

99. Sun C, Du H, Yin L, He M, Tian Y, Li H. Choice for the removal of bloody cerebrospinal fluid in postcoiling aneurysmal subarachnoid hemorrhage: external ventricular drainage or lumbar drainage? Turkish neurosurgery. 2014;24(5):737-44.

100. Tang QF, Lu SQ, Zhao YM, Qian JX. The changes of von willebrand factor/a disintegrin-like and metalloprotease with thrombospondin type I repeats-13 balance in aneurysmal subarachnoid hemorrhage. International Journal of Clinical and Experimental Medicine. 2015;8(1):1342-8.

101. Tanioka S, Ishida F, Nakano F, Kawakita F, Kanamaru H, Nakatsuka Y, et al. Machine Learning Analysis of Matricellular Proteins and Clinical Variables for Early Prediction of Delayed Cerebral Ischemia After Aneurysmal Subarachnoid Hemorrhage. Mol Neurobiol. 2019;56(10):7128-35.

102. Thampatty BP, Sherwood PR, Gallek MJ, Crago EA, Ren D, Hricik AJ, et al. Role of endothelin-1 in human aneurysmal subarachnoid hemorrhage: associations with vasospasm and delayed cerebral ischemia. Neurocrit Care. 2011;15(1):19-27.

103. Torne R, Hoyos J, Llull L, Rodriguez-Hernandez A, Munoz G, Mellado-Artigas R, et al. Edema resolution and clinical assessment in poor-grade subarachnoid hemorrhage: Useful indicators to predict delayed cerebral infarctions? Journal of Clinical Medicine. 2021;10(2):1-10.

104. Tseng MY, Hutchinson PJ, Kirkpatrick PJ. Effects of fluid therapy following aneurysmal subarachnoid haemorrhage: a prospective clinical study. British journal of neurosurgery. 2008;22(2):257-68.

105. van der Harst JJ, Luijckx G-JR, Elting JWJ, Bokkers RPH, van den Bergh WM, Eshghi OS, et al. Transcranial Doppler Versus CT-Angiography for Detection of Cerebral Vasospasm in Relation to Delayed Cerebral Ischemia After Aneurysmal Subarachnoid Hemorrhage: A Prospective Single-Center Cohort Study: The Transcranial doppler and CT-angiography for Investigating Cerebral vasospasm in Subarachnoid hemorrhage (TACTICS) study. Crit Care Explor. 2019;1(1):e0001-e.

106. Villain A, Boulouis G, Ben Hassen W, Rodriguez-Regent C, Trystram D, Edjlali M, et al. Small vessel disease in patients with subarachnoid hemorrhage: Prevalence and associations with vasospasm occurrence, severity and clinical outcomes. Neuroradiol. 2019;32(6):438-44.

107. Vrsajkov V, Jevdic J, Mihajlovic D, Pajtic V, Lazukic A, Pantic-Vrsajkov J. Ischemic Lesion on Computed Tomography after Subarachnoid Hemorrhage: Good Correlation with Angiographic Vasospasm and Worse Outcome. Neurosurgery Quarterly. 2016;26(3):225-9.

108. Wang JL, Li XN, Zhang YH, Li X, Yang YL. Evaluation function of transcranial two-dimensional and color Doppler ultrasonography (TCCS) for patients with different degrees of cerebral vasospasm before and after the nimodipine treatment. European review for medical and pharmacological sciences. 2017;21(11):2757-63.

109. Westermaier T, Stetter C, Vince GH, Pham M, Tejon JP, Eriskat J, et al. Prophylactic intravenous magnesium sulfate for treatment of aneurysmal subarachnoid hemorrhage: a randomized, placebo-controlled, clinical study. Critical care medicine. 2010;38(5):1284-90.

110. Williamson CA, Sheehan KM, Tipirneni R, Roark CD, Pandey AS, Thompson BG, et al. The Association Between Spontaneous Hyperventilation, Delayed Cerebral Ischemia, and Poor Neurological Outcome in Patients with Subarachnoid Hemorrhage. Neurocrit Care. 2015;23(3):330-8.

111. Woertgen C, Rothoerl RD, Albert R, Schebesch KM, Ullrich OW. Effects of temporary clipping during aneurysm surgery. Neurological Research. 2008;30(5):542-6.

112. Xu M, Luo L, Mao G. Effects of nimodipine combined with transcatheter arterial embolization intervention on cerebral aneurysm hemorrhage and prognosis. International Journal of Clinical and Experimental Medicine. 2020;13(10):7825-32.

113. Yang BH, He Q, Ding CY, Kang DZ, Tang QX. High-sensitivity C-reactive protein as a predictive factor of acute kidney injury following aneurysmal subarachnoid hemorrhage: a prospective observational study. Acta Neurochirurgica. 2019;161(9):1783-91.

114. Yang X, Peng J, Pang J, Wan W, Zhong C, Peng T, et al. The Association Between Serum Macrophage Migration Inhibitory Factor and Delayed Cerebral Ischemia After Aneurysmal Subarachnoid Hemorrhage. Neurotox Res. 2020;37(2):397-405.

115. Yoneda H, Shirao S, Nakagawara J, Ogasawara K, Tominaga T, Suzuki M. A prospective, multicenter, randomized study of the efficacy of eicosapentaenoic acid for cerebral vasospasm: the EVAS study. World Neurosurg. 2014;81(2):309-15.

116. Yoshimoto T, Shirasaka T, Fujimoto S, Yoshidumi T, Yamauchi T, Tokuda K, et al. Cilostazol may prevent cerebral vasospasm following subarachnoid hemorrhage. Neurologia Medico-Chirurgica. 2009;49(6):235-40.

117. Zhang C, Zhao S, Zang Y, Zhao W, Song Q, Feng S, et al. Magnesium sulfate in combination with nimodipine for the treatment of subarachnoid hemorrhage: a randomized controlled clinical study. Neurol Res. 2018;40(4):283-91.

118. Zhang H, Zhang B, Li S, Liang C, Xu K, Li S. Whole brain CT perfusion combined with CT angiography in patients with subarachnoid hemorrhage and cerebral vasospasm. Clin Neurol Neurosurg. 2013;115(12):2496-501.

119. Zhu Y, Jiang H, Li Y, Weng Y, Xu K, Zhou L, et al. Serum Alkaline Phosphatase Level is Associated with Angiographic Vasospasm, Delayed Cerebral Ischemia-Caused Clinical Deterioration, and Functional Outcome After Aneurysmal Subarachnoid Hemorrhage. Neurocrit Care. 2019;31(3):466-75.

120. Zwienenberg-Lee M, Hartman J, Rudisill N, Madden LK, Smith K, Eskridge J, et al. Effect of prophylactic transluminal balloon angioplasty on cerebral vasospasm and outcome in patients with fisher grade IIi subarachnoid hemorrhage: Results of a phase II multicenter, randomized, clinical trial. Stroke. 2008;39(6):1759-65.

Table S1: Characteristics of the included studies

| **Manuscript** | **Study Design** | **No. sites** | **No. participants** | **Mean age** $\boldsymbol{\pm}$ **SD** | **No. female participants (%)** | **No. high-grade WFNS or HH (%) *** | **No. high-grade Fisher (%)** $\boldsymbol{\dagger}$ | **Indication for vasospasm testing** | **Modality used to test for vasospasm** |
| --- | --- | --- | --- | --- | --- | --- | --- | --- | --- |
| Abla 2014 | PCS | 1 | 211 | 53.7$\pm$11.9 | 159 (75.3) | ND | ND | Routine | DSA, CTA |
| Acampa 2021 | PCS | 1 | 59 | 62$\pm$1 | 36 (61) | ND | 37 (62.7) | Routine | TCD, CTA |
| Akdemir 2009 | RCT | 1 | 83 | 53.7 | 51 (61.4) | 37 (44.6) | 32 (38.6) | Routine | TCD |
| Al-Mufti 2017 | PCS | 1 | 1286 | ND | 914 (71.1) | 386 (30) | 615 (47.8) | Routine | DSA, CTA |
| Anetsberger 2020 | RCT | 1 | 108 | ND | 82 (75.9) | 59 (54.6) | 74 (68.5) | Routine | TCD |
| Ateia 2020 | PCS | 1 | 50 | ND | 29 (58) | ND | 38 (76) | Routine | TCD |
| Bacigaluppi 2020 | PCS | 1 | 42 | 56.3$\pm$15.1 | 29 (69) | 10 (23.8) | 28 (66.7) | Routine | TCD, CTA, DSA |
| Barges-Coll 2013 | PCS | 1 | 40 | 47.3$\pm$2 | 22 (55) | 0 | 33 (82.5) | Routine | TCD |
| Bender 2020 | PCS | 1 | 292 | 51$\pm$1 | 170 (58.2) | ND | ND | Routine | TCD |
| Bergstrom 2013 | PCS | 1 | 48 | ND | 39 (81.3) | ND | 43 (89.6) | Symptoms | DSA |
| Borkar 2018 | RCT | 1 | 60 | 48 | 33 (55) | 34 (56.7) | ND | Routine | TCD |
| Bothun 2019 | PCS | 1 | 42 | 53$\pm$1 | 24 (57.1) | 18 (42.9) | 28 (66.7) | Routine | DSA, CTA, MRA |
| Bradford 2013 | RCT | 2 | 157 | 56.2 | 104 (68.2) | 47 (29.9) | ND | Routine | DSA |
| Budohoski 2012 | PCS | 1 | 98 | 56.7 | 69 (70.4) | 35 (35.7) | 67 (68.4) | Routine | DSA, TCD |
| Cagnazzo 2021 | PCS | 1 | 50 | 55.3$\pm$11.5 | 33 (66) | 33 (66) | 45 (90) | Routine | CTA |
| Carlson 2020 | RCT | 2 | 289 | 56$\pm$11 | 202 (69.9) | ND | 268 (92.7) | ND | ND |
| Chai 2011 | PCS | 1 | 85 | 48.5 | 46 (54.1) | 37 (43.5) | ND | Routine | CTA, DSA, TCD |
| Chang 2020 | PCS | 1 | 50 | 54$\pm2$ | ND | ND | ND | Routine | DSA, CTA |
| Chaudhry 2017 | PCS | 1 | 80 | 57 | 50 (62.5) | 51 (63.8) | 77 (96.3) | Symptoms | DSA, CTA, CTP |
| Chaudhry 2019 | PCS | 1 | 80 | 56.97$\pm$12 | 50 (62.5) | 51 (63.8) | 78 (97.5) | Routine | CTA, DSA |
| Chen 2020 | RCT | 3 | 317 | 75.66$\pm$7.9 | 164 (51.7) | ND | ND | Routine | TCD or DSA |
| Cheng 2016 | PCS | 1 | 58 | 52$\pm$11 | 33 (56.9) | 12 (20.7) | ND | ND | CTA |
| Chou 2010 | PCS | 1 | 189 | 53.7 | 136 (71.9) | 96 (50.8) | 157 (83.1) | Routine | TCD |
| Chou 2012 | PCS | 1 | 52 | 53.6 | 30 (57.7) | 26 (50) | 39 (75) | Routine | DSA |
| Chui 2020 | RCT | 1 | 40 | 58$\pm$11 | 31 (77.5) | 10 (25) | 24 (60) | ND | TCD, MRA or DSA |
| Cinotti 2019 | PCS | 1 | 208 | ND | 120 (57.7) | 109 (52.4) | 165 (79.3) | Routine | DSA, CTA |
| Constantinou 2021 | PCS | 1 | 60 | 56.5$\pm$10.7 | 39 (65) | 0 | ND | Symptoms | CTA, MRI |
| Ding 2015 | PCS | 1 | 79 | 45.9 | 32 (40.5) | 79 (100) | ND | ND | ND |
| Ding 2020 | PCS | 1 | 103 | 52.48$\pm$10.1 | 57 (55.3) | 56 (54.4) | ND | Routine | CTA, MRA |
| Djilvesi 2020 | PCS | 1 | 50 | 54.02$\pm$9.9 | 29 (58) | ND | 38 (76) | Routine | CTA |
| Duan 2018 | PCS | 132 | 504 | 57 | 295 (58.5) | 87 (17.3) | 202 (40.1) | Routine | TCD |
| Duris 2018 | PCS | 1 | 47 | 53.9 | 32 (68.1) | 34 (72.3) | 46 (97.9) | Routine | TCD |
| Ehrlich 2016 | PCS | 1 | 89 | 55.6 | 60 (67.4) | ND | ND | Routine | DSA, TCD |
| Ehrlich 2019 | PCS | 1 | 179 | ND | 113 (63.1) | 78 (43.6) | ND | Routine | DSA |
| Esmael 2020 | PCS | 1 | 40 | 51.1$\pm$1 | 22 (55) | 15 (37.5) | 23 (57.5) | Routine | DSA, CTA |
| Etminan 2013 | RCT | 1 | 60 | 56.1 | 38 (63.3) | 56 (93.3) | 58 (97.9) | Routine | DSA, CTA |
| Filipce 2015 | PCS | 1 | 224 | ND | 122 (54.5) | ND | ND | ND | ND |
| Fontanella 2008 | PCS | 1 | 786 | 55.3 | 458 (58.2) | 412 (52.4) | 623 (79.3) | TCD | DSA, TCD |
| Foreman 2016 | PCS | 2 | 153 | 54.8 | 115 (75.2) | 65 (42.5) | 129 (84.3) | Symptoms | DSA, CTA |
| Fountas 2009 | PCS | 3 | 41 | 51.8 | 16 (38.1) | 17 (41.5) | 14 (34.1) | Routine | DSA |
| Gallek 2013 | PCS | 1 | 235 | 53.4 | 165 (70.2) | 129 (54.9) | 161 (68.5) | Symptoms | DSA |
| Geng 2016 | RCT | 1 | 87 | 46.8 | 42 (48.3) | 44 (50.6) | 14 (16.1) | Routine | TCD |
| Gerner 2020 | PCS | 1 | 505 | 55.9$\pm$13.9 | 325 (64.3) | ND | ND | Routine | TCD |
| Gomis 2010 | RCT | 1 | 95 | 49.8 | 60 (63.2) | 32 (33.7) | 59 (62.1) | Symptoms | DSA, TCD |
| Goncalves 2019 | PCS | 1 | 149 | ND | 109 (73.2) | 54 (36.2) | 113 (75.8) | ND | DSA, TCD |
| Hattingen 2008 | PCS | 1 | 51 | 52.1 | 34 (66.7) | 25 (49) | 36 (70.6) | Routine | DSA |
| Haug Nordenmark 2019 | PCS | 1 | 51 | ND | 30 (58.8) | 11 (21.6) | 24 (47.1) | ND | CTA |
| Hostettler 2021 | PCS | 1 | 646 | 55.39$\pm$13 | 432 (66.9) | 258 (39.9) | 629 (97.4) | ND | DSA, CTA, MRA |
| Ianosi 2020 | PCS | 1 | 77 | ND | 56 (53.3) | ND | ND | ND | TCD |
| Inoue 2020 | PCS | 1 | 105 | 61.3 | 77 (73.3) | 56 (53.3) | ND | Routine | DSA |
| Jacquens 2020 | PCS | 1 | 398 | ND | 206 (51.8) | ND | 251 (63.1) | ND | DSA |
| Jafri 2020 | PCS | 1 | 61 | 56.4$\pm$14.6 | 46 (75.4) | ND | 38 (62.3) | Routine | DSA |
| Kanamaru 2020 | PCS | 9 | 238 | 64.37$\pm$14 | 169 (71) | 105 (44.1) | 202 (84.9) | ND | MRA, CTA |
| Karic 2017 | PCS | 1 | 156 | 55.6 | 134 (85.9) | 66 (42.3) | 96 (61.5) | Routine | CTA |
| Kasuya 2011 | PCS | 6 | 136 | ND | 87 (64) | 34 (25) | ND | Routine | DSA |
| Katagai 2021 | PCS | 1 | 178 | 61$\pm$13 | 133 (74.7) | 88 (49.4) | ND | Routine | DSA |
| Kim 2018 | PCS | 1 | 87 | 59.6 | 56 (64.4) | 46 (52.9) | 60 (69) | Routine | DSA |
| Kim 2014 | PCS | 1 | 121 | 59.6 | 71 (58.7) | 74 (61.2) | 83 (68.6) | Routine | DSA |
| Kim 2008 | PCS | 1 | 45 | 52.9$\pm$12.7 | ND | ND | ND | ND | DSA |
| Kimura 2014 | PCS | 2 | 130 | 65.3$\pm$13.7 | 98 (75.4) | 72 (55.4) | ND | Routine | DSA, CTA |
| Konar 2019 | PCS | 1 | 100 | 51.6 | 58 (58) | 24 (24) | 85 (85) | ND | ND |
| Kronvall 2009 | RCT | 1 | 106 | 56.2 | 81 (76.4) | 51 (48.1) | 95 (89.6) | Routine | TCD |
| Kumar 2017 | PCS | 1 | 421 | 51.5 | 269 (64) | 264 (62.7) | 183 (43.5) | Routine | DSA |
| Kuroi 2020 | PCS | 1 | 291 | 63.3 | 194 (67) | ND | ND | Routine | CTA |
| Lai 2019 | PCS | 1 | 44 | 58 | 39 (88.6) | 22 (50) | 40 (90.9) | Symptoms | DSA |
| Lee 2020 | RCT | 1 | 46 | 54.59$\pm$11.8 | 34 (73) | 21 (45.7) | 44 (95.7) | ND | DSA |
| Li 2018 | PCS | 1 | 90 | ND | 42 (46.7) | 51 (56.7) | 45 (50) | Routine | DSA, TCD |
| Llull 2021 | PCS | 1 | 131 | ND | 87 (66.4) | 0 | 103 (78.6) | Routine | TCD, DSA |
| Macdonald 2008 | RCT | 52 | 96 § | 52 | 64 (66.7) | 30 (31.3) | 78 (81.3) | Routine | DSA |
| Maduri 2019 | PCS | 9 | 264 | 55.8 | 180 (68.2) | 142 (53.8) | 239 (90.5) | ND | ND |
| Malinova 2016 | PCS | 1 | 753 | 53.3 | 496 (65.9) | 264 (35.1) | 540 (71.7) | Routine | TCD |
| Matsuda 2016 | RCT | 3 | 148 | 58.5 | 100 (67.6) | 63 (42.6) | 147 (99.3) | Routine | DSA |
| Moritz 2010 | PCS | 1 | 55 | 54 | 39 (70.1) | 20 (36.4) | 52 (94.5) | Routine | TCD |
| Moussouttas 2014 | PCS | 1 | 57 | 55 | 38 (66.7) | 15 (26.3) | 31 (54.4) | Routine | TCD |
| Nakagawa 2013 | PCS | 1 | 103 | 63.5 | 68 (66) | 41 (39.8) | 57 (55.3) | Symptoms or TCD | DSA |
| Naraoka 2018 | RCT | 4 | 54 | 55 | 34 (63) | 19 (35.2) | 52 (96.3) | Routine | DSA |
| Ni 2011 | PCS | 1 | 46 | 54 | 22 (47.8) | 7 (15.2) | 28 (60.9) | Routine | TCD |
| Ohnishi 2013 | PCS | 1 | 95 | 62.2 | 53 (55.8) | 39 (41.1) | 86 (90.5) | Routine | DSA |
| Olson 2013 | RCT | 1 | 60 | 58.1 | 43 (71.7) | ND | ND | Symptoms and TCD | DSA |
| Opancina 2020 | PCS | 1 | 66 | 53.5$\pm$18.9 | 48 (72.7) | ND | ND | ND | DSA |
| Ozono 2020 | PCS | 38 | 1124 | 62.4$\pm$13.5 | 772 (68.7) | 490 (43.6) | 910 (81) | ND | CT |
| Park 2015 | PCS | 1 | 234 | 55.8 | 141 (60.3) | 134 (57.3) | 156 (66.7) | Symptoms or TCD | DSA, TCD |
| Ramesh 2014 | PCS | 1 | 208 | 50.5 | 113 (54.3) | 50 (24) | ND | ND | ND |
| Rasmussen 2016 | PCS | 1 | 90 | 53 | 74 (82.2) | 13 (14.4) | 90 (100) | Routine | CTA |
| Reynolds 2020 | PCS | 1 | 345 | 54.5$\pm$13.4 | 234 (67.8) | 240 (69.6) | 310 (89.9) | Routine | TCD, CTA, MRI |
| Ridwan 2019 | PCS | 1 | 101 | ND | 70 (69.3) | 56 (55.5) | 67 (66.3) | Routine | TCD |
| Roederer 2014 | PCS | 1 | 81 | 54 | 57 (70.4) | 60 (74.1) | 78 (96.3) | Symptoms | DSA, TCD |
| Rynkowski 2019 | PCS | 1 | 40 | 53.9$\pm$12.1 | 28 (70) | ND | 37 (92.5) | Routine | TCD |
| Sanchez-Pena 2008 | PCS | 1 | 109 | 49.2 | 74 (67.9) | 42 (38.5) | 88 (80.7) | Symptoms | DSA, TCD |
| Sanelli 2011 | PCS | 1 | 75 | 48 | 52 (69.3) | 30 (40) | ND | Symptoms or TCD | DSA |
| Schebesch 2013 | PCS | 1 | 66 | 52.8 | 40 (60.6) | 48 (72.7) | ND | Routine | TCD |
| Schmidt 2008 | PCS | 1 | 576 | 53.3 | 380 (66) | 324 (56.3) | ND | Symptoms or TCD | DSA, TCD |
| Schneider 2011 | PCS | 1 | 54 | 53.2 | ND | 29 (53.7) | ND | Routine | DSA |
| Shikata 2019 | PCS | 4 | 55 | 64.6$\pm$12.1 | 41 (74.5) | ND | 15 (27.3) | Symptoms | DSA |
| Staalso 2013 | PCS | 1 | 111 | 55 | 81 (73) | 52 (46.8) | 105 (94.6) | Symptoms | DSA |
| Starke 2008 | PCS | 1 | 77 | 54.2 | 49 (63.6) | 33 (42.9) | 42 (54.6) | Symptoms or CT or MR perfusion | DSA |
| Suarez 2012 | PCS | 6 | 47 | 51 | 34 (72.3) | 6 (12.8) | 41 (87.2) | Routine | TCD |
| Sugimoto 2018 | RCT | 1 | 27 | 64.8 | 19 (70.4) | 9 (33.3) | 24 (88.9) | Routine | DSA |
| Sun 2014 | RCT | 1 | 148 | 56.8 | 75 (50.7) | 148 (100) | ND | Symptoms or TCD | DSA |
| Tang 2015 | PCS | 1 | 58 | 53 | 34 (58.6) | 30 (51.7) | 22 (37.9) | Routine | TCD |
| Tanioka 2019 | PCS | 7 | 95 | ND | 66 (69.5) | 38 (40) | 88 (92.6) | Routine | DSA, CTA |
| Thampatty 2011 | PCS | 1 | 106 | 53.9 | 75 (70.8) | 76 (23.9) | 80 (75.5) | Symptoms | DSA |
| Torne 2021 | PCS | 1 | 47 | 59.28$\pm$12.9 | 33 (70.2) | 47 (100) | ND | ND | DSA, CTA |
| Tseng 2009 | PCS | 1 | 160 | 55.4 | 100 (62.5) | 61 (38.1) | 141 (88.1) | Routine | TCD |
| van der Harst 2019 | PCS | 1 | 59 | 57$\pm$11 | 37 (62.7) | 11 (18.6) | 53 (89.8) | Routine | CTA |
| Villain 2019 | PCS | 1 | 113 | ND | 70 (61.9) | 27 (23.9) | 90 (79.6) | Routine | TCD |
| Vrsajkov 2016 | PCS | 1 | 54 | 54.3 | 31 (57.4) | ND | ND | Routine | CTA |
| Wang 2017 | PCS | 1 | 77 | 37.2 | 21 (27.2) | ND | ND | Routine | TCD |
| Westermaier 2010 | RCT | 1 | 107 | 51 | 41 (38.3) | 62 (57.9) | 73 (68.2) | Symptoms or TCD | TCD, CTA |
| Williamson 2015 | PCS | 1 | 207 | 57.8 | 141 (68.1) | ND | ND | Symptoms or TCD | DSA |
| Woertgen 2008 | PCS | 1 | 292 | 50.6 | 188 (64.4) | 87 (29.8) | 179 (61.3) | Routine | TCD |
| Xu 2020 | RCT | 1 | 65 | 58.1$\pm$12.8 | 31 (47.7) | 9 (13.9) | ND | ND | TCD |
| Yang 2019 | PCS | 1 | 164 | 53.3$\pm$10.5 | 90 (54.9) | 82 (50) | ND | ND | CTA, MRA |
| Yang 2020 | PCS | 1 | 201 | ND | 112 (55.7) | ND | ND | Routine | TCD |
| Yoneda 2014 | RCT | 5 | 162 | 59.3 | 109 (67.3) | 45 (27.8) | 137 (84.6) | Routine | TCD |
| Yoshimoto 2009 | PCS | 1 | 24 | 58 | 14 (58.3) | 8 (33.3) | 20 (83.3) | Routine | DSA |
| Zhang 2018 | RCT | 1 | 120 | 43 | 58 (48.3) | 51 (42.5) | ND | Routine | TCD |
| Zhang 2013 | PCS | 1 | 60 | 53.5 | 32 (53.3) | 8 (13.3) | 22 (36.7) | Routine | CTA, DSA, TCD |
| Zhu 2019 | PCS | 1 | 196 | 57.5$\pm$10.3 | 103 (52.6) | ND | 102 (52) | ND | DSA |
| Zwienenberg-Lee 2008 | RCT | 10 | 170 | 55 | 111 (65.3) | 104 (61.2) | 170 (100) | Routine | TCD |

*WFNS or HH 4-5; $\dagger$Fisher 3 or 4, or equivalent; § only control group included in analysis as intervention arm has well established effect on vasospasm incidence; ND: not described; RCT: Randomised Controlled Trial; PCS: Prospective cohort study

Figure S1: Risk of Bias assessment for included Randomised Controlled Trials (Cochrane ROB2 tool)


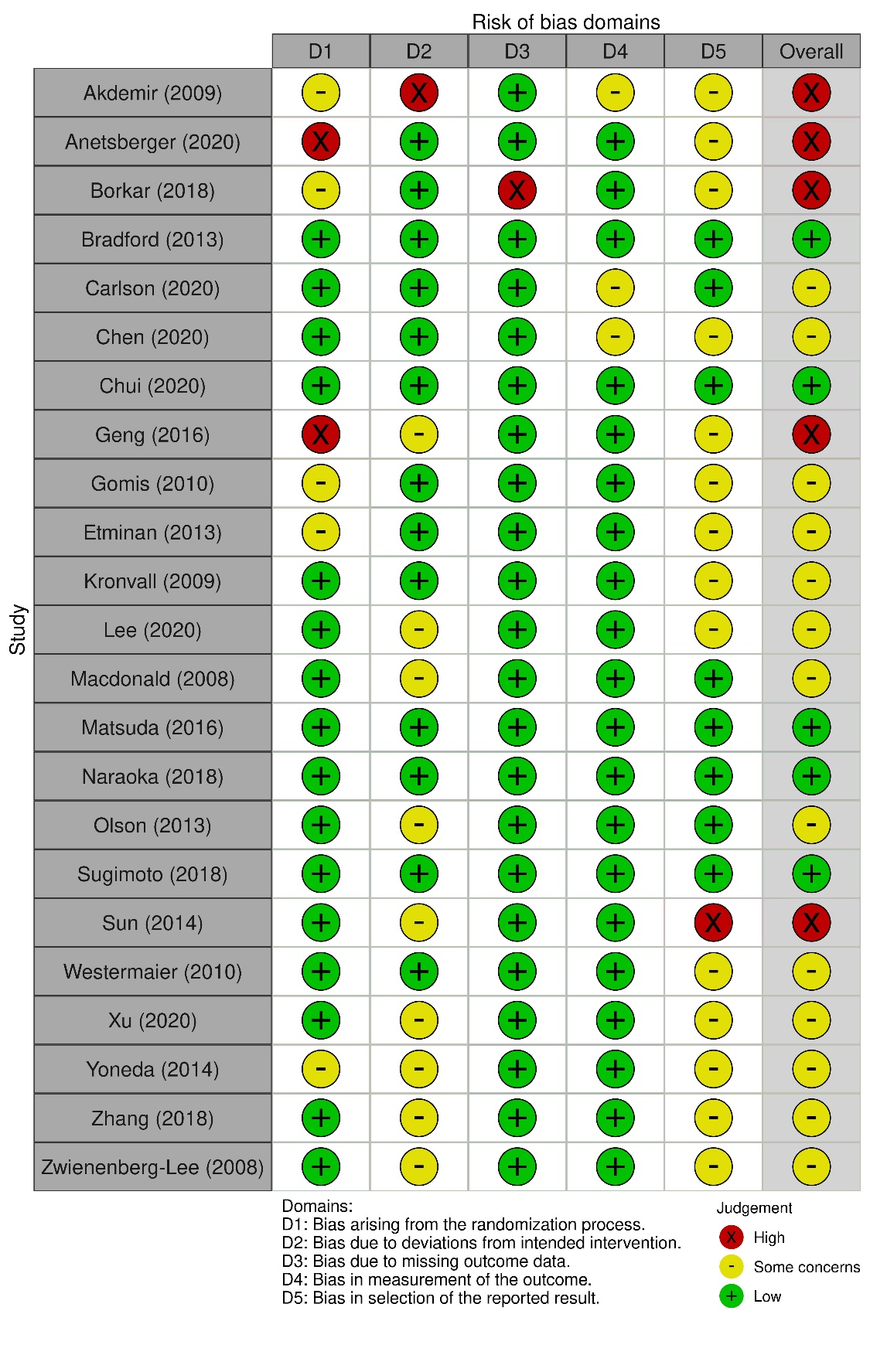


Table S2: Risk of Bias assessment for included prospective cohort studies (Newcastle-Ottawa Scale)

| Author (Year) | Cohort Selection Score  (Maximum 4) | Comparability of exposed and non-exposed cohorts  (Maximum 2) | Outcome assessment score  (Maximum 3) | Total Score (Maximum 9) |
| --- | --- | --- | --- | --- |
| Abla (2014) | ★★★★ |  | ★★★ | 7 |
| Acampa (2021) | ★★★ |  | ★★ | 5 |
| Al-Mufti (2016) | ★★★★ | ★ | ★★★ | 8 |
| Ateia (2020) | ★★★★ |  | ★★ | 6 |
| Bacigaluppi (2020) | ★★★★ |  | ★★★ | 7 |
| Barges-Coll (2013) | ★★★★ |  | ★★★ | 7 |
| Bender (2020) | ★★★ |  | ★★★ | 6 |
| Bergstrom (2013) | ★★★★ |  | ★★★ | 7 |
| Bothun (2019) | ★★★ |  | ★★ | 5 |
| Budohoski (2012) | ★★★★ | ★ | ★★ | 7 |
| Cagnazzo (2021) | ★★★★ |  | ★★ | 6 |
| Chai (2011) | ★★★ | ★ | ★★ | 6 |
| Chang (2020) | ★★★★ |  | ★★★ | 7 |
| Chaudhry (2017) | ★★★ | ★ | ★★ | 6 |
| Chaudhry (2019) | ★★★★ |  | ★★★ | 7 |
| Cheng (2016) | ★★★★ |  | ★★ | 6 |
| Chou (2010) | ★★★★ | ★ | ★★ | 7 |
| Chou (2012) | ★★★★ | ★ | ★★ | 7 |
| Cinotti (2019) | ★★★★ |  | ★★★ | 7 |
| Constantinou (2021) | ★★★★ |  | ★★★ | 7 |
| Ding (2015) | ★★ |  |  | 2 |
| Ding (2020) | ★★★ |  | ★★★ | 6 |
| Djilvesi (2020) | ★★★ |  | ★★ | 5 |
| Duan (2018) | ★★★★ |  | ★ | 5 |
| Duris (2018) | ★★★ |  | ★★ | 5 |
| Ehrlich (2016) | ★★★ | ★ | ★★★ | 7 |
| Ehrlich (2019) | ★★★ |  | ★★★ | 6 |
| Esmael (2020) | ★★ | ★ | ★★★ | 6 |
| Filipce (2015) | ★★ |  |  | 2 |
| Fontanella (2008) | ★★★★ | ★ | ★★★ | 8 |
| Foreman (2016) | ★★★★ | ★ | ★ | 6 |
| Fountas (2009) | ★★★★ |  | ★★ | 6 |
| Gallek (2013) | ★★★★ |  | ★ | 5 |
| Gerner (2020) | ★★★★ |  | ★★ | 6 |
| Goncalves (2019) | ★★ |  | ★★ | 4 |
| Hattingen (2008) | ★★★★ |  | ★★ | 6 |
| Haug Nordenmark (2019) | ★★ |  | ★★★ | 5 |
| Hostettler (2021) | ★★★ |  | ★★ | 5 |
| Ianosi (2020) | ★★★ |  | ★★ | 5 |
| Inoue (2013) | ★★★★ | ★ | ★★ | 7 |
| Jacquens (2020) | ★★★ |  | ★★ | 5 |
| Jafri (2020) | ★★★★ |  | ★★★ | 7 |
| Kanamaru (2020) | ★★★ |  | ★★ | 5 |
| Karic (2017) | ★★★★ |  | ★★ | 6 |
| Kasuya (2011) | ★★★★ |  | ★★ | 6 |
| Katagai (2021) | ★★★★ |  | ★★ | 6 |
| Kim (2018) | ★★★★ |  | ★★ | 6 |
| Kim (2014) | ★★★★ |  | ★★ | 6 |
| Kim (2008) | ★★★★ |  | ★★★ | 7 |
| Kimura (2014) | ★★★★ |  | ★★★ | 7 |
| Konar (2019) | ★★★ |  | ★★ | 5 |
| Kumar (2017) | ★★★★ |  | ★★★ | 7 |
| Kuroi (2020) | ★★★★ |  | ★★ | 6 |
| Lai (2019) | ★★★★ |  | ★★★ | 7 |
| Li (2008) | ★★★★ |  | ★ | 5 |
| Llull (2021) | ★★★★ |  | ★★★ | 7 |
| Maduri (2019) | ★ | ★ | ★★★ | 5 |
| Malinova (2016) | ★★★★ | ★ | ★★ | 7 |
| Moritz (2010) | ★★★★ | ★ | ★★★ | 8 |
| Moussouttas (2014) | ★★★★ | ★ | ★ | 6 |
| Nakagawa (2013) | ★★★★ | ★ | ★★ | 7 |
| Ni (2011) | ★★★ |  | ★★ | 5 |
| Ohnishi (2013) | ★★★★ | ★ | ★★★ | 8 |
| Opancina (2020) | ★★★★ |  | ★★★ | 7 |
| Ozono (2020) | ★★★★ |  | ★★★ | 7 |
| Park (2015) | ★★★★ | ★ | ★★★ | 8 |
| Ramesh (2014) | ★★★ | ★ | ★★ | 6 |
| Rasmussen (2016) | ★★★★ | ★ | ★★★ | 8 |
| Reynolds (2020) | ★★★★ | ★ | ★★ | 7 |
| Ridwan (2019) | ★★★ |  | ★★★ | 6 |
| Rynkowski (2019) | ★★★★ |  | ★★★ | 7 |
| Roederer (2014) | ★★★★ | ★ | ★★ | 7 |
| Sanchez-Pena (2008) | ★★★★ | ★ | ★★★ | 8 |
| Sanelli (2011) | ★★★★ | ★ | ★★★ | 8 |
| Schebesch (2013) | ★★★ | ★ | ★★ | 6 |
| Schmidt (2008) | ★★★★ |  | ★★ | 6 |
| Schneider (2011) | ★★★★ | ★ | ★★★ | 8 |
| Shikata (2019) | ★★★★ |  | ★★★ | 7 |
| Staalso (2013) | ★★★★ | ★ | ★★★ | 8 |
| Starke (2008) | ★★★★ | ★ | ★★★ | 8 |
| Suarez (2012) | ★★★★ |  | ★★ | 6 |
| Tanioka (2019) | ★★★ |  | ★★★ | 6 |
| Tang (2015) | ★★★ |  | ★★★ | 6 |
| Thampatty (2011) | ★★★★ | ★ | ★★★ | 8 |
| Torne (2021) | ★★★ |  | ★★★ | 6 |
| Tseng (2009) | ★★★★ | ★ | ★★★ | 8 |
| Villain (2019) | ★★★ |  | ★★★ | 6 |
| van der Harst (2019) | ★★★★ |  | ★★★ | 7 |
| Vrsajkov (2016) | ★★★★ | ★ | ★★★ | 8 |
| Wang (2017) | ★★ | ★ | ★★ | 5 |
| Williamson (2015) | ★★★★ |  | ★★★ | 7 |
| Woertgen (2008) | ★★★ | ★ | ★★★ | 7 |
| Yang (2019) | ★★★★ | ★ | ★★★ | 8 |
| Yang (2020) | ★★★★ | ★ | ★★★ | 8 |
| Yoshimoto (2009) | ★★★★ | ★ | ★★ | 7 |
| Zhang (2013) | ★★★★ | ★ | ★★ | 7 |
| Zhu (2019) | ★★★★ | ★ | ★★★ | 8 |

Table S3: Criteria used to define arterial vasospasm in the included studies

| Definition | Included studies |
| --- | --- |
| Angiography – DSA and/or CTA |  |
| Angiographic evidence of intracranial arterial narrowing to any degree | Abla 2014; Al-Mufti 2017; Bergstrom 2013; Bradford 2013; Bothun 2019; Budohoski 2012; Cagnazzo 2021; Chai 2011; Chen 2020; Chui 2020; Cinotti 2019; Ding 2020; Djilvesi 2020; Ehrlich 2019; Esmael 2020; Foreman 2016; Goncalves 2019; Hostettler 2021; Inoue 2013; Jacquens 2020; Karic 2017; Kasuysa 2011; Katagai 2021; Kumar 2017; Lai 2019; Lee 2020; Li 2018; Sanelli 2011; Staalso 2013; Starke 2008; van der Harst 2019; Williamson 2015; Zhang 2013; Yang 2019 |
| Angiographic evidence of intracranial arterial narrowing to any degree and symptoms | Bergstrom 2013; Gomis 2010; Nakagawa 2012 |
| > 10% reduction in vessel calibre | Ehrlich 2016; Hattingen 2008 |
| Decreased arterial diameter > 20% | Zhu 2019; Llull 2021 |
| Decreased arterial diameter > 25% | Fontanella 2008; Gallek 2013; Kim 2014; Matsuda 2016; Naraoka 2018; Olson 2013; Thampatty 2011; |
| Decreased arterial diameter of >/= 25% and symptoms of neurological deterioration | Donnelly 2015 |
| Decreased arterial diameter of >30% | Etminan 2013; Westermaier 2010 |
| Decreased arterial diameter of >/=33% | Schneider 2011 |
| Decreased arterial diameter of >33% | MacDonald 2008; Sugimoto 2018; Vrsajkov 2016 |
| Decreased arterial diameter of >34% | Opancina 2020 |
| Decreased arterial diameter of >50% | Chaudry 2017; Chou 2012; Kanamaru 2020; Kim 2018; Kimura 2014; Kuroi 2020; Ohnishi 2013; Tanioka 2019; Yoshimoto 2009; |
| Angiography, no further details given | Chang 2020; Choudry 2019; Constantinou 2021; Nordenmark 2019; Jafri 2020; Kim 2008; Konar 2019; Rasmussen 2016; Reynolds 2020; Roederer 2014; Schmidt 2008; Shikata 2019; Sun 2014; |
| CT Perfusion |  |
| Mean transit time of >5 seconds or >2 seconds when compared to the contralateral side on CTP | Chaudry 2017 |
| 1.5- fold prolongation of reference mean transit time values from healthy controls | Etminan 2013 |
| Transcranial Doppler |  |
| Any accelerated flow and symptoms of neurological deterioration | Gomis 2010 |
| Flow velocity >160m/s and symptoms | Akdemir 2009; |
| MCA peak velocity > 200cm/s | Chai 2011; Chen 2020; Zhang 2013 |
| MCA mean velocity >120 cm/s | Anetsberger 2020; Ateia 2020; Bacigaluppi 2020; Bender 2020; Chai 2011; Chou 2010; Chui 2020; Duris 2018; Esmael 2020; Fontanella 2008; Gerner 2020; Geng 2016; Ianosi 2020; Kronvall 2018; Li 2018; Llul 2021; Malinova 2016; Sanchez-Pena 2008; Schmidt 2008; Sun 2014; Tang 2015; Wang 2017; Woertgen 2008; Yoneda 2014; Zhang 2018; Zhang 2013; Ridwan 2019 |
| MCA mean velocity >140cm/s | Westermaier 2010 |
| MCA mean velocity >150cm/s | Park 2014; Schebesch 2013 |
| MCA mean velocity >180cm/s | Yoshimoto 2009 |
| MCA mean velocity > 200 cm/s | MacDonald 2008; |
| ACA mean velocity > 90 cm/s | Esmael 2020; |
| ACA mean velocity > 100 cm/s | Duris 2018; |
| ACA mean velocity >120 cm/s | Anetsberger 2020; Ianosi 2020; Fontanella 2008; Moussouttas 2014; Yoneda 2014 |
| ACA mean velocity > 150 cm/s | Schebesch 2013 |
| ACA mean velocity >180cm/s | Yoshimoto 2009 |
| ACA mean velocity > 200 cm/s | MacDonald 2008; |
| ACA mean velocity > 90 cm/s with a concomitant Lindegaard Ratio above 3.0 | Villain 2019; |
| MCA flow velocity >120 cm/s with a concomitant Lindegaard Ratio above 3.0 | Budohoski 2012; Duan 2018; Foreman 2016; MacDonald 2008; Moussouttas 2014; Rynkowksi 2019; Tseng 2009; Villain 2019; Yang 2020; Goncalves 2019; |
| MCA or ACA velocity > 130 cm/s with a Lindegaard ratio greater  than 3.0. | Olson 2013; Zwienenberg-Lee 2008 |
| Vertebrobasilar mean flow >80cm/s with a modified Lindegaard ratio greater than 2.0. | Villain 2019; |
| Vertebrobasilar mean flow >60cm/s | Esmael 2020; |
| Vertebrobasilar mean flow >80cm/s | Tang 2015; |
| Vertebrobasilar mean flow >90cm/s | Westermaier 2010; Yoneda 2014 |
| Flow velocity >120cm/s, vessel not specified | Moritz 2010 |
| Lindegaard ratio > 3.0 with symptoms and infarction on CT | Barges-Coll 2013; |
| Lindegaard ratio > 3.0 in anterior circulation | Chen 2020; Park 2015; Tang 2015; Reynolds 2020; |
| Lindegaard ratio > 2.0 in posterior circulation | Reynolds 2020 |
| Increase in flow velocity of >50cm/s on previous measurement | Anetsberger 2020; MacDonald 2008; Moritz 2010; Park 2015; Sanchez-Pena 2008; Yoshimoto 2009 |
| Increased flow velocities, not otherwise specified | Acampa 2021; Borkar 2018; Ni 2011 |
| Other |  |
| Not described | Carlson 2020; Cheng 2016; Ding 2015; Filipce 2015; Fountas 2009; Maduri 2019; Ozono 2020; Ramesh 2014; Suarez 2012. Torné 2021; Xu 2020 |

**Table S4: Estimated pooled incidence of cerebral arterial vasospasm following aneurysmal subarachnoid haemorrhage by year of publication.**

| Year | N | Estimate of incidence of aVSP | 95% CI | *I^2^* |
| --- | --- | --- | --- | --- |
| 2008 | 9 | 0.38 | 0.28 to 0.48 | 96.2% |
| 2009 | 5 | 0.41 | 0.32 to 0.51 | 72.6% |
| 2010 | 4 | 0.57 | 0.36 to 0.78 | 95.7% |
| 2011 | 6 | 0.37 | 0.26 to 0.48 | 85.0% |
| 2012 | 3 | 0.36 | 0.14 to 0.58 | - |
| 2013 | 11 | 0.36 | 0.27 to 0.46 | 91.9% |
| 2014 | 9 | 0.37 | 0.25 to 0.49 | 95.6% |
| 2015 | 5 | 0.28 | 0.15 to 0.41 | 95.4% |
| 2016 | 8 | 0.38 | 0.26 to 0.51 | 95.4% |
| 2017 | 5 | 0.56 | 0.31 to 0.80 | 99.0% |
| 2018 | 8 | 0.51 | 0.28 to 0.73 | 98.5% |
| 2019 | 17 | 0.42 | 0.31 to 0.52 | 96.7% |
| 2020 | 24 | 0.48 | 0.40 to 0.55 | 96.7% |
| 2021 | 7 | 0.41 | 0.30 to 0.52 | 92.5% |

N = number of studies, aVSP= cerebral arterial vasospasm, CI = confidence intervals.

Figure S2: The pooled estimated incidence of cerebral arterial vasospasm following aneurysmal subarachnoid haemorrhage: fixed effect model (sensitivity analysis)


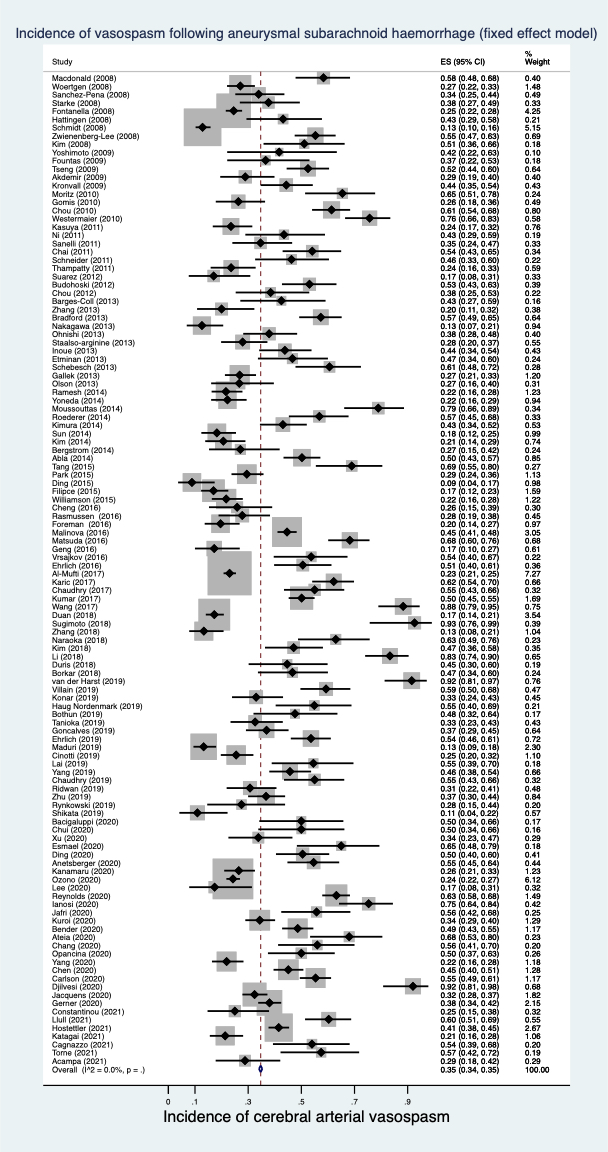


Figure S3: The pooled estimated incidence of cerebral arterial vasospasm following aneurysmal subarachnoid haemorrhage: by study type (random effects model)


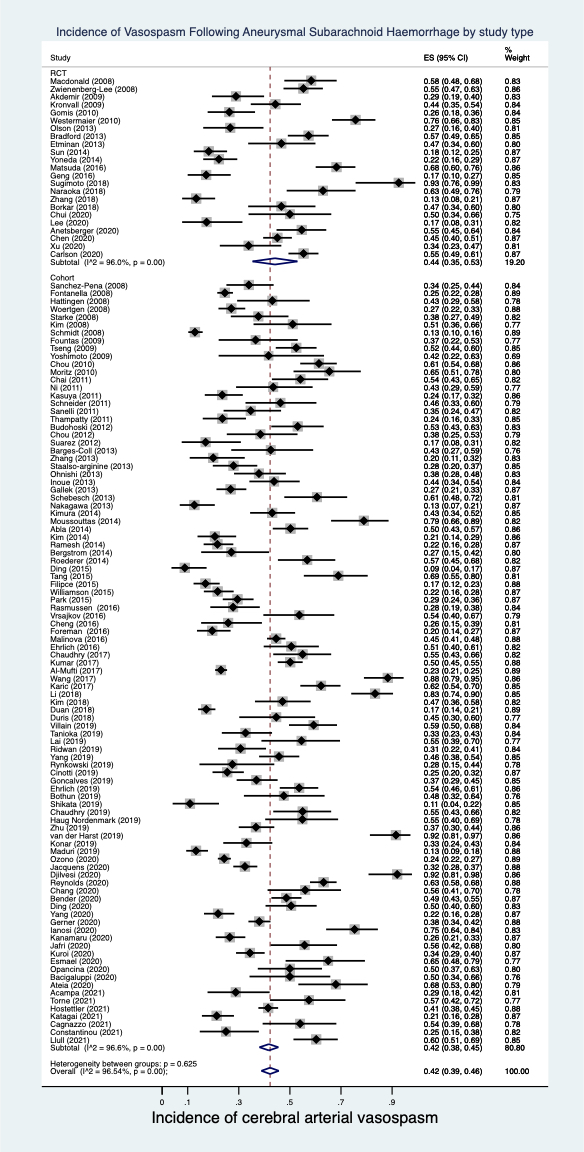


Figure S4: The pooled estimated incidence of cerebral arterial vasospasm following aneurysmal subarachnoid haemorrhage: by mode of investigation (random effects model)


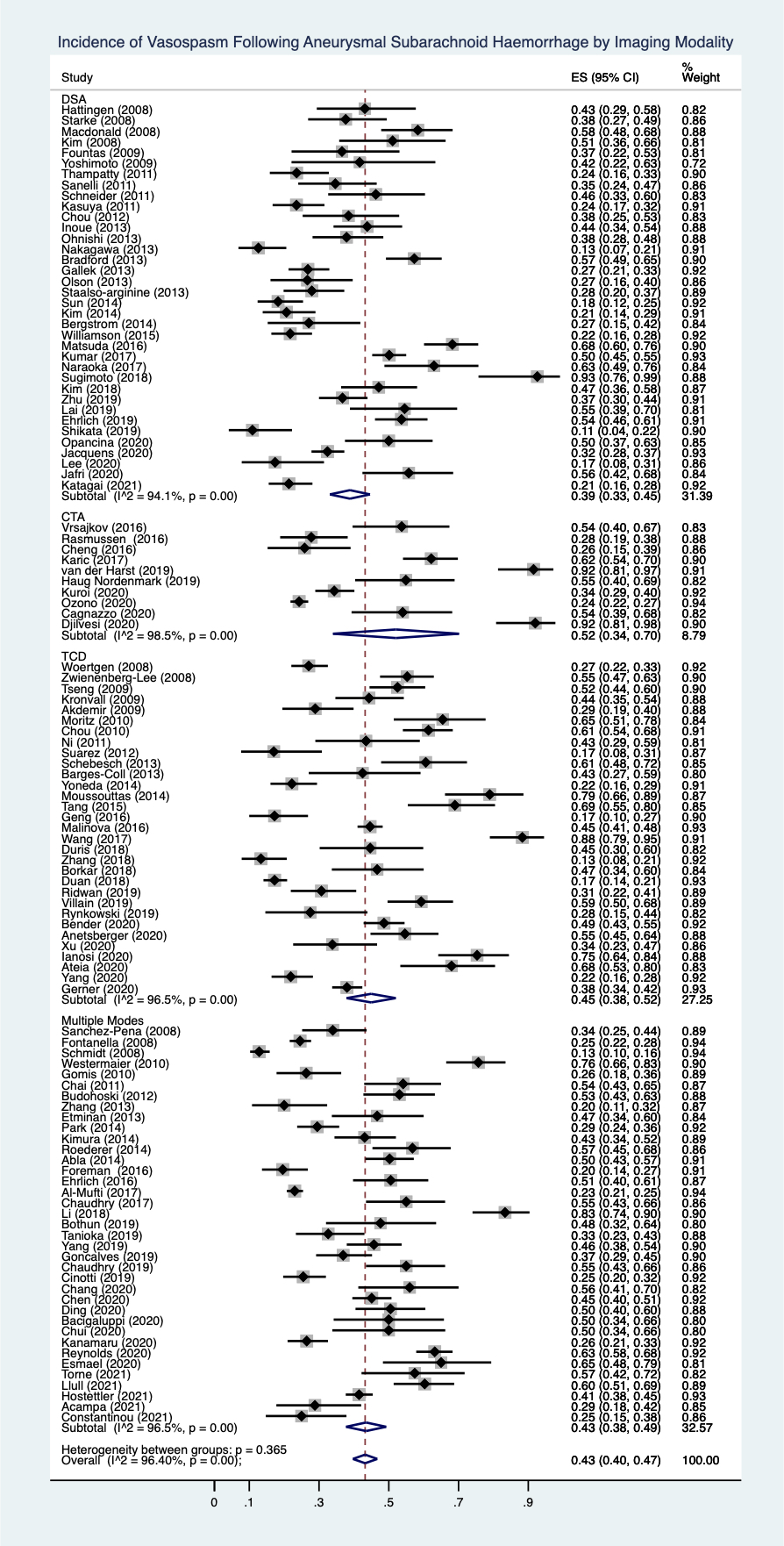


Figure S5: The pooled estimated incidence of cerebral arterial vasospasm following aneurysmal subarachnoid haemorrhage: indication for imaging (random effects model)


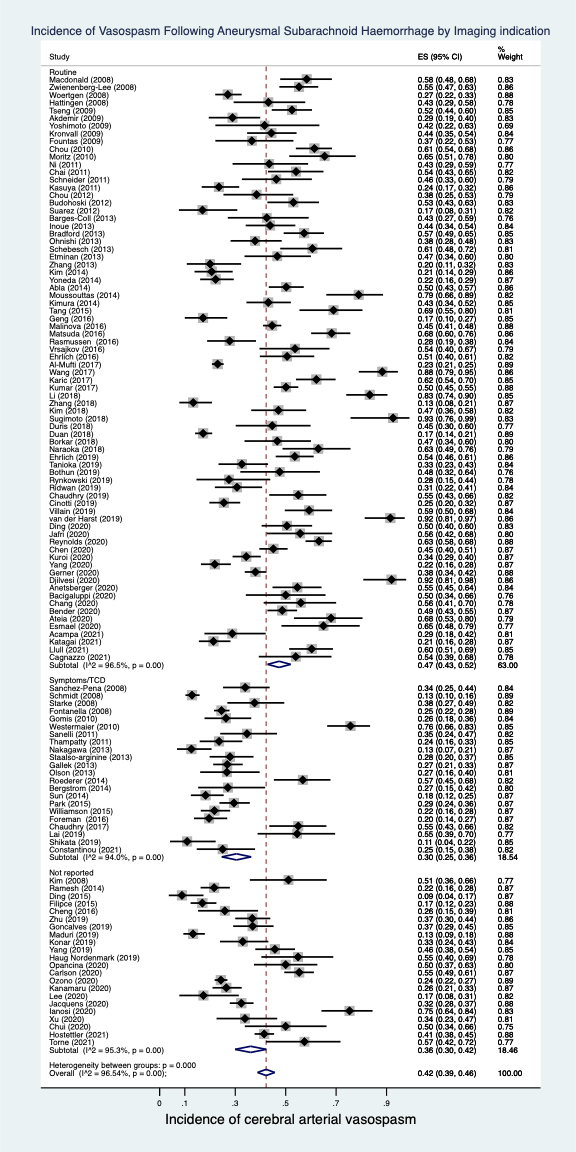


Figure S6: The pooled estimated incidence of cerebral arterial vasospasm following aneurysmal subarachnoid haemorrhage in studies performing routine DSA (random effects model)


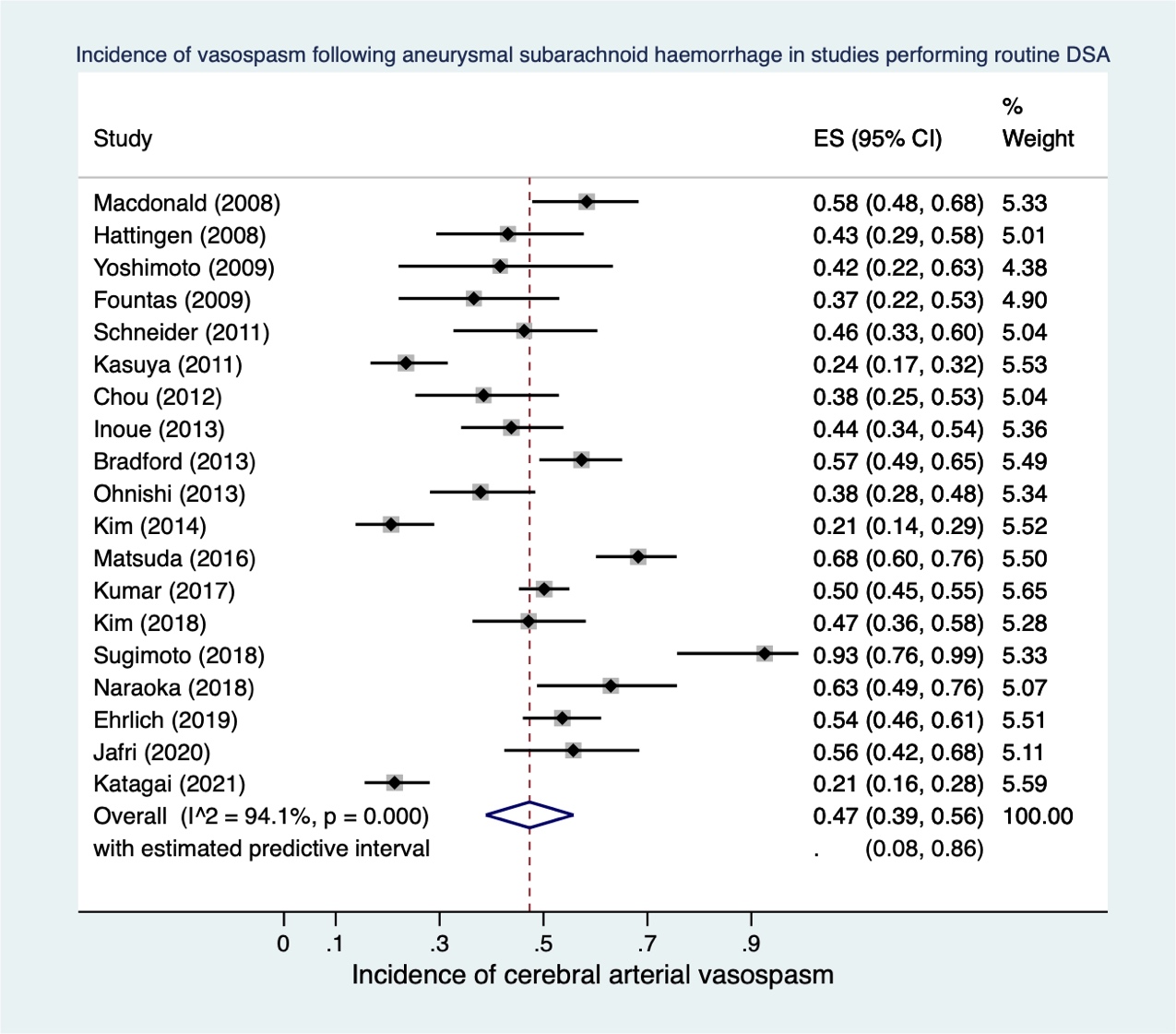


Figure S7: The pooled estimated incidence of cerebral arterial vasospasm following aneurysmal subarachnoid haemorrhage: by proportion of study participants with high grade CT scans (random effects model)


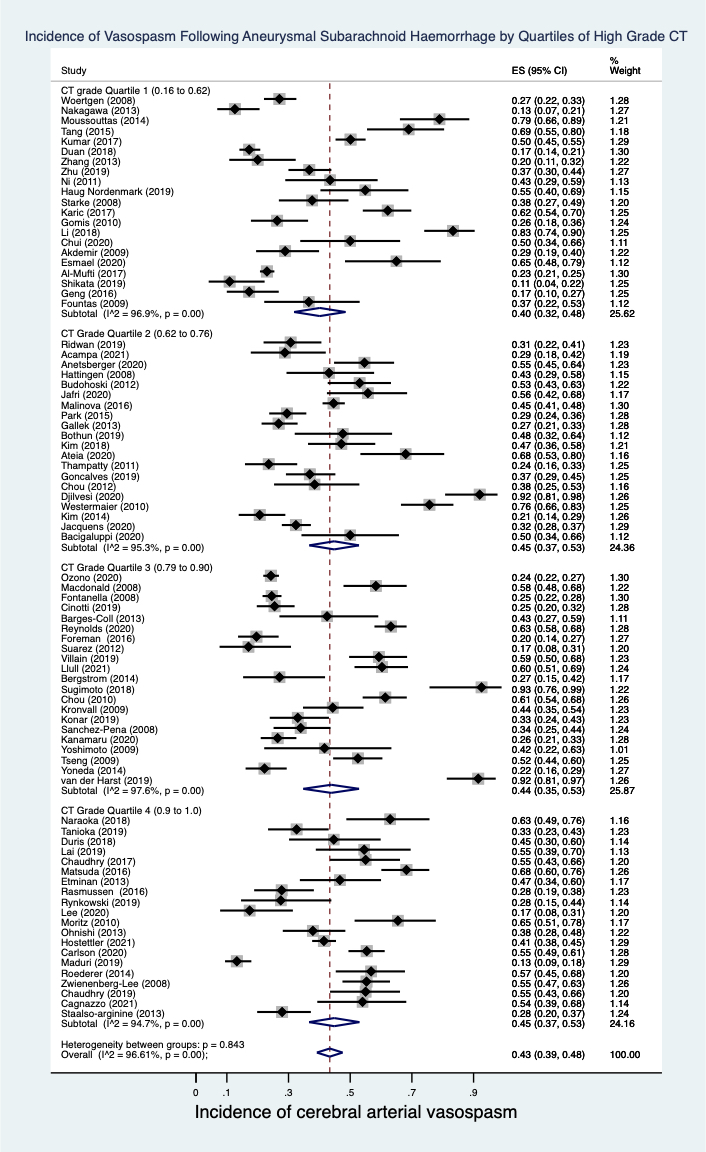


Figure S8: The pooled estimated incidence of cerebral arterial vasospasm following aneurysmal subarachnoid haemorrhage: by proportion of study participants with poor clinical grade at presentation (random effects model)


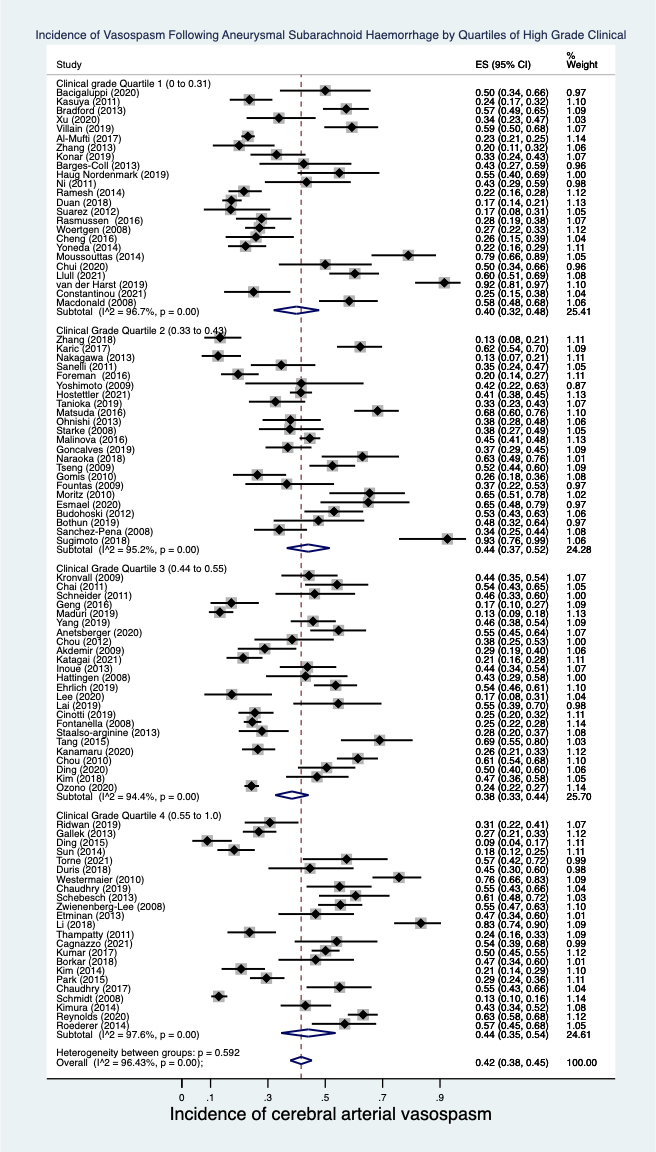


Figure S9: The pooled estimated incidence of cerebral arterial vasospasm by year (random effects model)

Figure S10: The pooled estimated incidence of delayed cerebral ischaemia following aneurysmal subarachnoid haemorrhage (random effects model)


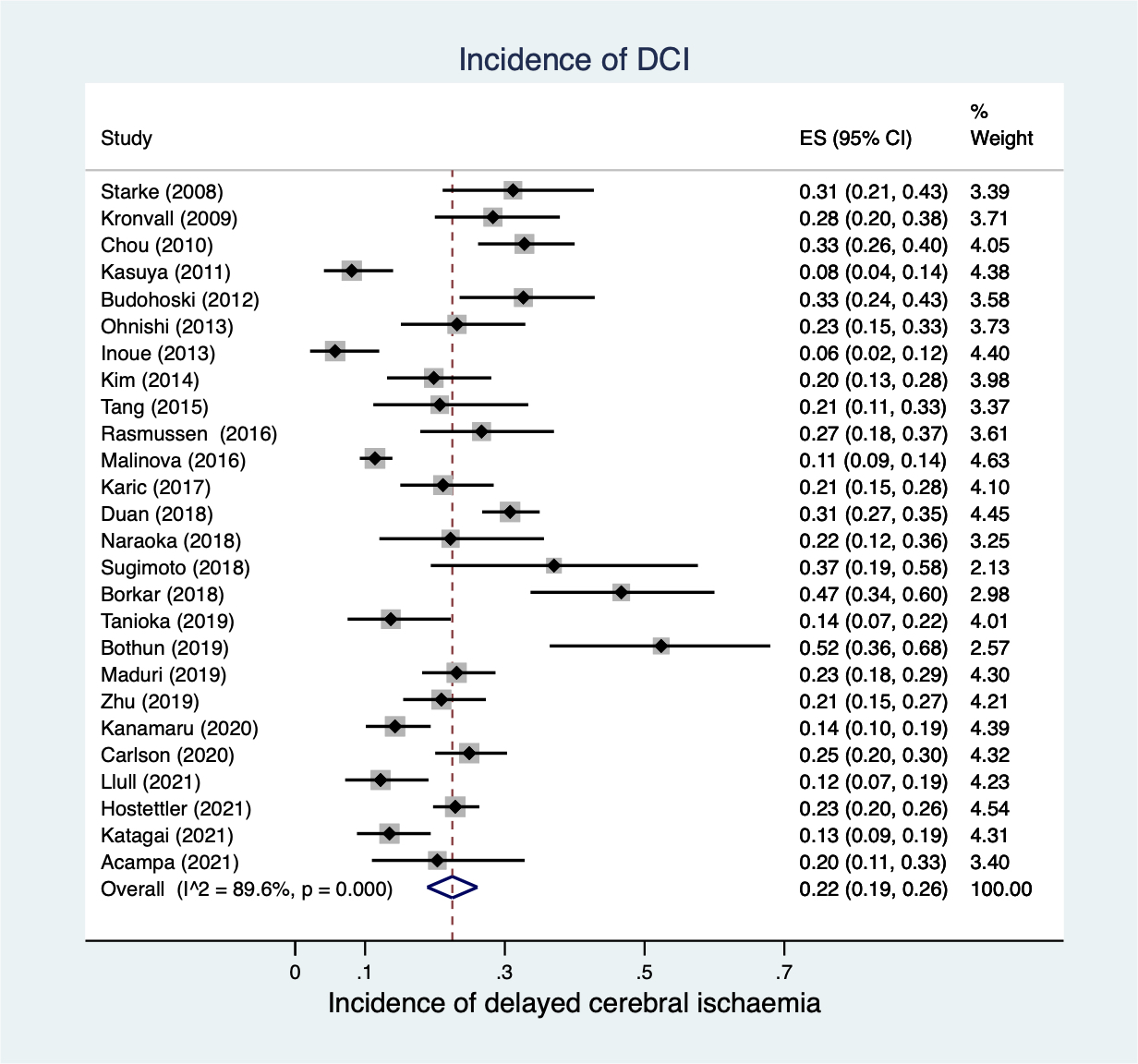


Search Strategy

MEDLINE – OVID

| **#** | **Searches** |
| --- | --- |
| 1 | exp Subarachnoid Hemorrhage/ |
| 2 | exp INTRACRANIAL ANEURYSM/ |
| 3 | exp Aneurysm, Ruptured/ |
| 4 | exp Vasospasm, Intracranial/ |
| 5 | exp Brain Ischemia/ |
| 6 | exp Brain Infarction/ |
| 7 | cerebral ischemia.mp. |
| 8 | cerebral ischaemia.mp. |
| 9 | delayed cerebral ischaemia.mp. |
| 10 | delayed cerebral ischemia.mp. |
| 11 | exp Cerebral Infarction/ |
| 12 | 4 or 5 or 6 or 7 or 8 or 9 or 10 or 11 |
| 13 | 1 or 2 or 3 |
| 14 | 12 and 13 |
| 15 | Humans/ |
| 16 | 14 and 15 |
| 17 | limit 16 to (english language and ("therapy (maximizes sensitivity)" or "diagnosis (best balance of sensitivity and specificity)")) |

| **#** | **Searches** |
| --- | --- |
| 1 | exp Subarachnoid Hemorrhage/ |
| 2 | exp INTRACRANIAL ANEURYSM/ |
| 3 | exp ANEURYSM, RUPTURED/ |
| 4 | exp Vasospasm, Intracranial/ |
| 5 | exp Brain Ischemia/ |
| 6 | exp Brain Infarction/ |
| 7 | cerebral ischemia.mp. |
| 8 | cerebral ischaemia.mp. |
| 9 | delayed cerebral ischaemia.mp. |
| 10 | delayed cerebral ischemia.mp. |
| 11 | exp Cerebral Infarction/ |
| 12 | 4 or 5 or 6 or 7 or 8 or 9 or 10 or 11 |
| 13 | 1 or 2 or 3 |
| 14 | 12 and 13 |
| 15 | randomized controlled trial.pt. |
| 16 | controlled clinical trial.pt. |
| 17 | randomized.ab,ti. |
| 18 | placebo.ab,ti. |
| 19 | drug therapy.sh. |
| 20 | randomly.ab,ti. |
| 21 | trial.ab,ti. |
| 22 | groups.ab,ti. |
| 23 | 15 or 16 or 17 or 18 or 19 or 20 or 21 or 22 |
| 24 | exp Animals/ |
| 25 | exp Humans/ |
| 26 | 24 not 25 |
| 27 | 23 not 26 |
| 28 | 27 and 14 |

| **#** | **Searches** |
| --- | --- |
| 1 | exp Subarachnoid Hemorrhage/ |
| 2 | exp INTRACRANIAL ANEURYSM/ |
| 3 | exp Aneurysm, Ruptured/ |
| 4 | exp Vasospasm, Intracranial/ |
| 5 | exp Brain Ischemia/ |
| 6 | exp Brain Infarction/ |
| 7 | cerebral ischemia.mp. |
| 8 | cerebral ischaemia.mp. |
| 9 | delayed cerebral ischaemia.mp. |
| 10 | delayed cerebral ischemia.mp. |
| 11 | exp Cerebral Infarction/ |
| 12 | 4 or 5 or 6 or 7 or 8 or 9 or 10 or 11 |
| 13 | 1 or 2 or 3 |
| 14 | 12 and 13 |
| 15 | exp Cohort Studies/ |
| 16 | exp Case-Control Studies/ |
| 17 | comparative study.pt. |
| 18 | exp Risk Factors/ |
| 19 | cohort.tw. |
| 20 | compared.tw. |
| 21 | groups.tw. |
| 22 | case control.tw. |
| 23 | multivariate.tw. |
| 24 | 15 or 16 or 17 or 18 or 19 or 20 or 21 or 22 or 23 |
| 25 | 24 and 14 |

EMBASE

| **#** | **Searches** |
| --- | --- |
| 1 | exp subarachnoid hemorrhage/ |
| 2 | exp intracranial aneurysm/ |
| 3 | exp aneurysm rupture/ |
| 4 | exp brain vasospasm/ |
| 5 | exp brain ischemia/ |
| 6 | exp brain infarction/ |
| 7 | cerebral ischaemia.mp. |
| 8 | cerebral ischemia.mp. |
| 9 | delayed cerebral ischaemia.mp. |
| 10 | delayed cerebral ischemia.mp. |
| 11 | cerebral infarction.mp. |
| 12 | 4 or 5 or 6 or 7 or 8 or 9 or 10 or 11 |
| 13 | 1 or 2 or 3 |
| 14 | 12 and 13 |
| 15 | crossover procedure/ |
| 16 | double-blind procedure/ |
| 17 | randomized controlled trila / |
| 18 | single-blind procedure/ |
| 19 | (random* or factorial* or crossover* or cross over* or placebo* or doubl* blind* or singl* blind* or assign* or allocate* or volunteer*).ab,ti. |
| 20 | 15 or 16 or 17 or 18 or 19 |
| 21 | 14 and 20 |

| **#** | **Searches** |
| --- | --- |
| 1 | exp subarachnoid hemorrhage/ |
| 2 | exp intracranial aneurysm/ |
| 3 | exp aneurysm rupture/ |
| 4 | exp brain vasospasm/ |
| 5 | exp brain ischemia/ |
| 6 | exp brain infarction/ |
| 7 | cerebral ischaemia.mp. |
| 8 | cerebral ischemia.mp. |
| 9 | delayed cerebral ischaemia.mp. |
| 10 | delayed cerebral ischemia.mp. |
| 11 | cerebral infarction.mp. |
| 12 | 4 or 5 or 6 or 7 or 8 or 9 or 10 or 11 |
| 13 | 1 or 2 or 3 |
| 14 | 12 and 13 |
| 15 | exp clinical article/ |
| 16 | exp controlled study/ |
| 17 | exp major clinical study/ |
| 18 | exp prospective study/ |
| 19 | exp cohort analysis/ |
| 20 | cohort.ab,ti. |
| 21 | compared.ab,ti. |
| 22 | groups.ab,ti. |
| 23 | case control.ab,ti. |
| 24 | multivariate.ab,ti. |
| 25 | or/15-24 |
| 26 | 14 and 25 |

PubMED

Search (((((aneurysm ruptured) OR intracranial aneurysm) OR subarachnoid haemorrhage)) AND ((((delayed cerebral ischaemia) OR brain infarction) OR brain ischaemia) OR vasospasm intracranial)) AND (((((clinical[Title/Abstract]) AND trial[Title/Abstract])) OR ((((((((clinical[Title/Abstract]) AND trial[Title/Abstract])) OR clinical trials[MeSH Terms]) OR clinical trial[Publication Type]) OR random*[Title/Abstract]) OR random allocation[MeSH Terms]) OR therapeutic use[MeSH Subheading])) OR (((((cohort study) OR follow up studies) OR comparative studies) OR incidence studies) OR prospective cohort study))

Search (((((aneurysm, ruptured[MeSH Terms]) OR aneurysms, intracranial[MeSH Terms]) OR hemorrhage, subarachnoid[MeSH Terms])) AND ((((((((intracranial vasospasm[MeSH Terms]) OR brain ischemia[MeSH Terms]) OR brain infarction[MeSH Terms]) OR cerebral ischaemia) OR cerebral ischemia) OR delayed cerebral ischaemia) OR delayed cerebral ischemia) OR cerebral infarction[MeSH Terms])) AND ((((((((((cohort studies[MeSH Terms]) OR case control studies[MeSH Terms]) OR comparative study[Publication Type]) OR risk factors[MeSH Terms]) OR cohort[Text Word]) OR compared[Text Word]) OR groups[Text Word]) OR case control[Text Word]) OR multivariate[Text Word]) AND ( "2008/01/01"[PDat] : "2019/12/31"[PDat] ) AND Humans[Mesh])

Search (((((aneurysm, ruptured[MeSH Terms]) OR aneurysms, intracranial[MeSH Terms]) OR hemorrhage, subarachnoid[MeSH Terms])) AND ((((((((intracranial vasospasm[MeSH Terms]) OR brain ischemia[MeSH Terms]) OR brain infarction[MeSH Terms]) OR cerebral ischaemia) OR cerebral ischemia) OR delayed cerebral ischaemia) OR delayed cerebral ischemia) OR cerebral infarction[MeSH Terms])) AND ((((((((randomized controlled trial [pt]) OR controlled clinical trial [pt]) OR randomized [tiab]) OR placebo [tiab]) OR drug therapy [sh]) OR randomly [tiab]) OR trial [tiab]) OR group
